# Transcriptomic and Network Analysis of Minor Salivary Glands of Patients With Primary Sjögren’s Syndrome

**DOI:** 10.3389/fimmu.2020.606268

**Published:** 2021-01-08

**Authors:** Akinsola Oyelakin, Erich Horeth, Eun-Ah Christine Song, Sangwon Min, Monika Che, Brandon Marzullo, Christopher J. Lessard, Astrid Rasmussen, Lida Radfar, R. Hal Scofield, David M. Lewis, Donald U. Stone, Kiely Grundahl, Scott S. De Rossi, Zoya Kurago, A. Darise Farris, Kathy L. Sivils, Satrajit Sinha, Jill M. Kramer, Rose-Anne Romano

**Affiliations:** ^1^ Department of Oral Biology, State University of New York at Buffalo, Buffalo, NY, United States; ^2^ Genomics and Bioinformatics Core, State University of New York at Buffalo, Buffalo, NY, United States; ^3^ Arthritis and Clinical Immunology, Oklahoma Medical Research Foundation, Oklahoma City, OK, United States; ^4^ College of Dentistry, University of Oklahoma Health Sciences Center, Oklahoma City, OK, United States; ^5^ Department of Medicine, University of Oklahoma Health Sciences Center, Oklahoma City, OK, United States; ^6^ Department of Veteran’s Affairs Medical Center, Oklahoma City, OK, United States; ^7^ Dean McGee Eye Institute, University of Oklahoma Health Sciences Center, Oklahoma City, OK, United States; ^8^ Dental College of Georgia, Augusta University, Augusta, GA, United States; ^9^ Department of Biochemistry, State University of New York at Buffalo, Buffalo, NY, United States

**Keywords:** Sjögren’s syndrome, salivary gland, RNA-sequencing, gene expression, bioinformatics

## Abstract

Primary Sjögren’s syndrome (pSS) is a systemic autoimmune disease characterized primarily by immune-mediated destruction of exocrine tissues, such as those of the salivary and lacrimal glands, resulting in the loss of saliva and tear production, respectively. This disease predominantly affects middle-aged women, often in an insidious manner with the accumulation of subtle changes in glandular function occurring over many years. Patients commonly suffer from pSS symptoms for years before receiving a diagnosis. Currently, there is no effective cure for pSS and treatment options and targeted therapy approaches are limited due to a lack of our overall understanding of the disease etiology and its underlying pathology. To better elucidate the underlying molecular nature of this disease, we have performed RNA-sequencing to generate a comprehensive global gene expression profile of minor salivary glands from an ethnically diverse cohort of patients with pSS. Gene expression analysis has identified a number of pathways and networks that are relevant in pSS pathogenesis. Moreover, our detailed integrative analysis has revealed a primary Sjögren’s syndrome molecular signature that may represent important players acting as potential drivers of this disease. Finally, we have established that the global transcriptomic changes in pSS are likely to be attributed not only to various immune cell types within the salivary gland but also epithelial cells which are likely playing a contributing role. Overall, our comprehensive studies provide a database-enriched framework and resource for the identification and examination of key pathways, mediators, and new biomarkers important in the pathogenesis of this disease with the long-term goals of facilitating earlier diagnosis of pSS and to mitigate or abrogate the progression of this debilitating disease.

## Introduction

Sjögren’s syndrome (SS) is a chronic, inflammatory autoimmune disease typically characterized by focal lymphocytic infiltration of exocrine glands that predominantly affect the salivary and lacrimal glands, resulting in oral and ocular dryness, respectively. While this disease preferentially involves salivary and lacrimal glands, systemic effects are also observed. Indeed, a wide range of other affected organs include the skin, kidney, and lungs (1). Sjögren’s syndrome may exist as an independent entity, referred to as primary Sjögren’s syndrome (pSS), or alternatively as secondary Sjögren’s syndrome, which occurs in conjunction with other autoimmune connective tissue diseases such as rheumatoid arthritis or systemic lupus erythematosus (SLE). The prevalence of pSS ranges from 0.01% to 3% of the general population, primarily affecting middle-aged women with a female to male ratio of up to 20:1 (2, 3). While pSS is overwhelmingly dominated by ocular and oral dryness, fatigue, and pain, serious disease sequelae associated with increased mortality are observed, such as cryoglobulinemic vasculitis, B cell lymphoma and pulmonary fibrosis (4).

pSS is a multifactorial complex disease involving both genetic and environmental factors such as viral infections, that may influence disease progression and severity (5, 6). Viruses have long been considered potential players in SS pathology with Epstein-Barr and human T cell leukemia type I viruses being the most commonly associated with this disease (7–11). Over the years, studies also focused on the contribution of different immune cell types to the development of SS including T and B cells (12–14). More recently, these studies have expanded to examine the intimate role of signaling molecules and pathways in SS pathogenesis including various cytokines and chemokines. Indeed, multiple chemokines have been implicated in SS, including CCL19, CCL21, and CXCL13, all of which have been shown to play important roles in driving the immune-related effects associated with this disease (15–17). In addition to cytokines and chemokines, several signaling pathways are implicated in SS pathogenesis. Interestingly, emerging evidence suggests a prominent role for interferon (IFN) signaling in SS pathology (18). Indeed, activated IFN signaling in the salivary gland of SS patients has been associated with this disease (17, 19). Despite extensive efforts directed towards identifying the underlying molecular mechanisms contributing to SS pathogenesis, current treatment options are limited to managing clinical symptoms as no effective treatments, or cures, have been developed to date.

In order to better understand the underlying molecular landscape contributing to SS pathogenesis, we have performed RNA-sequencing to examine the global gene expression profiles of minor salivary glands (MSGs) from non-SS controls and pSS patients. Functional gene enrichment and network analysis of the MSGs revealed important molecular players and pathways that are relevant in pSS pathology. Moreover, our integrated transcriptomic analysis has uncovered a distinct pSS molecular signature highlighted by pertinent genes, genetic biomarkers and pathways that may be important in driving this disease. Furthermore, we show that the cellular dysfunction in the MSGs of pSS involves intricate contribution of various immune cells types, with epithelial cells also playing a potentially important role. Overall, our comprehensive studies have not only re-affirmed the importance of key signaling molecules and pathways, but have also identified novel genes and important cellular subtypes. This knowledge can be mined for effective diagnosis and monitoring of pSS pathology with the long-term goal of developing targeted therapeutic strategies to better treat this chronic debilitating disease.

## Materials and Methods

### Patients Samples

Human minor salivary glands (MSG) from 10 pSS patients and 10 non-SS controls were collected at the Oklahoma Medical Research Foundation (OMRF) as previously described (20). An additional three pSS patients and three non-SS controls were collected at the Augusta University Dental College of Georgia (AUDCG) (**Supplementary Table 5**). The OMRF and the AUDGC Institutional Review Boards approved all research procedures and the study participants gave written informed consent in compliance with the Declaration of Helsinki. All primary Sjögren’s syndrome patients were classified as either pSS or non-SS in accordance with the 2002 revised American European Consensus Group Sjögren’s syndrome criteria (AECG) (21). Patients also met the 2016 ACR-EULAR criteria for pSS (22, 23). The non-SS controls were subjects with subjective sicca symptoms, based on positive responses to the AECG standardized dry eyes and dry mouth questions, but who did not have the objective criteria of glandular dysfunction to be classified as pSS. MSG biopsy tissue from female pSS patients (n = 13, mean age = 55.1 years) and age and sex-matched non-SS control subjects (n = 13, mean age = 54.2 years) were analyzed. All pSS patients displayed focal lymphocytic sialadenitis with a focus score of greater than one/4 mm^2^ in accordance with the standard of care as detailed in Daniels et al. (24). A majority of patients exhibited Ro/SSA autoantibodies and salivary gland hypofunction (whole unstimulated salivary flow < 0.1 ml/min). A summary of clinical characteristics and patient demographics is provided in **Table 1** and **Supplementary Table S5**.

**Table 1:** A Summary of Clinical Characteristics and Patient Demographics.

Patient ID (sample ID)	Group	Age	Sex	Ethnicity	SSA	SSB	Focus score	Whole saliva flow (<0.1 ml/min)
p1032179-8(S1)	Non-SS	51	F	White/NativeAm	–	–	0	–
p1033491-4(S2)	Non-SS	58	F	Unknown	–	–	0	–
p1033567-0(S3)	Non-SS	52	F	White	–	–	0	–
p1033570-8(S4)	Non-SS	48	F	White	–	–	0	–
p1033749-8(S5)	Non-SS	64	F	White	–	–	0	–
p1033790-4(S6)	Non-SS	51	F	White	–	–	0	–
p1034307-9(S7)	Non-SS	64	F	White	–	–	0	–
p1034335-9(S8)	Non-SS	52	F	White	–	–	0	–
p1000509-3(S11)	SS	50	F	White	+	+	5	+
p1000529-6(S12)	SS	52	F	White	+	+	6.4	+
p1001613-4(S14)	SS	68	F	NativeAm	+	+	12	+
p1001703-6(S15)	SS	59	F	White/NativeAm	+	+	6	+
p1012568-8(S16)	SS	47	F	White/NativeAm	+	+	10.6	+
p1025909-5(S17)	SS	51	F	White	+	+	10	+
p1031753-1(S18)	SS	51	F	White	+	+	2.2	+
p1034325-7(S19)	SS	57	F	Unknown	+	+	5.5	+
p1034535-4(S20)	SS	61	F	White	+	+	8	+

### RNA Isolation and Quantitative RT-PCR

Total RNA from human MSGs from non-SS controls and pSS patient samples from OMRF was extracted from optimal cutting temperature (OCT) embedded tissues and isolated and purified using TRIzol (Invitrogen, 15596018) with BioMashers (TaKaRa, 9790A). Total RNA from human MSGs from non-SS controls and pSS patient samples from CDMUA was similarly isolated and purified using TRIzol with BioMashers. All RNA was phase separated by chloroform and further isolated using the Direct-zol RNA Miniprep kit (Zymo Research, R2050) according to the manufacturer’s instructions. For quantitative reverse-transcription polymerase chain reaction (qRT-PCR) a total of 0.8 micrograms of RNA was reverse transcribed using the iScript cDNA Synthesis Kit (Bio-Rad, 1708890) according to the manufacturer’s instructions. Quantitative reverse-transcription polymerase chain reaction was performed on a CFX96 Touch™ Real-Time PCR Detection System (Bio-Rad, 1855195) using iQ SYBR Green Supermix (Bio-Rad, 1708882). All qRT-PCR assays were performed in duplicates in at least three independent experiments. Relative expression values of each target gene were normalized to glyceraldehyde 3-phosphate dehydrogenase (GAPDH) expression. The RNA samples used for qRT-PCR analysis shown in **Supplementary Figure S2** represent three non-SS controls (S2, S4, and S5) and three pSS (S12, S14, and S16) collected from the OMRF. In parallel, an additional set of four patient samples were utilized consisting of three non-SS controls (S21-S23) and three pSS (S25-S27) collected from the CDMUA as well as one non-SS control (S24) and one pSS (S28) from the OMRF **Supplementary Figure S3**. These patient samples serve as an independent cohort for the qRT-PCR data since these samples were not processed for RNA-seq studies. Primer sequences are provided in **Supplementary Table S1**.

### RNA-Sequencing, Differentially Expressed Gene (DEG), Ingenuity Pathway Analysis (IPA), and Enrichment Analyses

**Figure 1:**
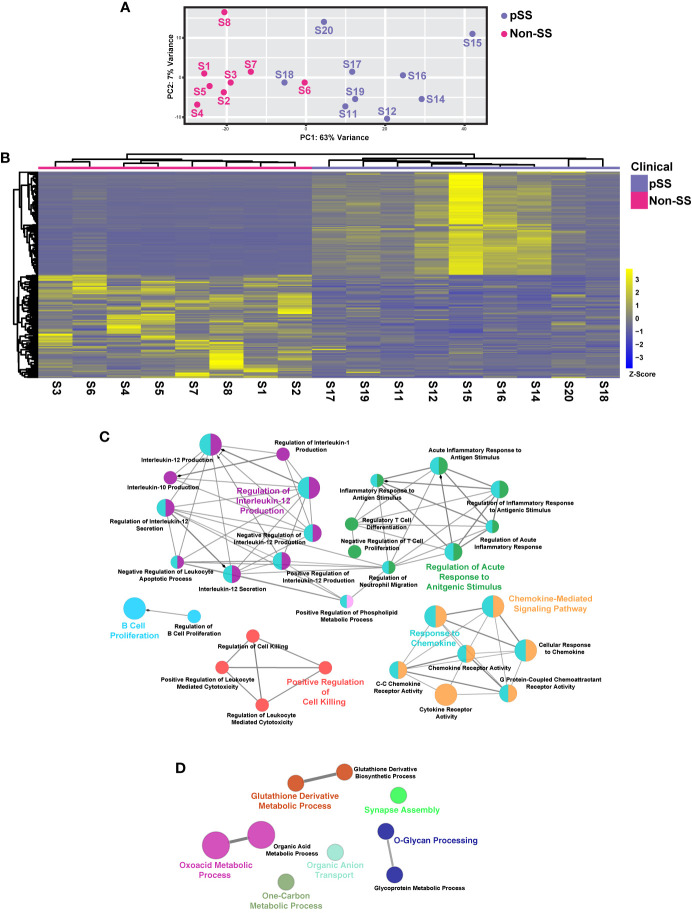
Comprehensive Transcriptomic Analysis of Minor Salivary Glands of Patients with Sjögren’s Syndrome. **(A)** Plot demonstrates principal component analysis (PCA) coordinates for each minor salivary gland for eight non-pSS controls and nine pSS patient samples. Blue and red circles represent pSS and non-pSS, respectively. **(B)** Heatmap visualization of the top 100 differentially expressed genes (DEGs) in minor salivary glands of non-SS controls and pSS patient samples. Network visualization of enriched biological processes of the top 100 upregulated **(C)** and top 100 downregulated **(D)** DEGs in pSS.

cDNA libraries were prepared using the TrueSeq RNA Sample Preparation Kit (Illumina) from RNA samples isolated from eight non-SS controls and nine pSS patients obtained from the OMRF (see **Figure 1A** and **Table 1** for sample ID numbers and additional patient information). The cDNA libraries were then sequenced on an Illumina NovaSeq sequencer (50 cycle paired-end). After initial quality control metrics were determined using FASTQC v0.4.3, the raw reads were then mapped to the reference genome (Hg38 build) with Hisat2 (25) v2.1.0. using Bowtie (26) v2.2.6 as the underlying aligner. Reads mapping uniquely to each gene in the reference genome were then quantified using featureCounts (27) from the Subread (28) package. The resulting counts matrix was imported into R for read count normalization and differential gene expression analysis using the DESeq2 (29) package. An adjusted p-value < 0.05 based on Benjamini-Hochberg method was used as a cut-off for determining differentially expressed genes. Gene ontology analysis for identification of enriched pathways was performed using the Database for Analysis Visualization and Integrated Discovery (DAVID) v6.8 (30, 31). The databases were queried by providing the list of official names of genes of interest. The resulting KEGG Pathway (32–35) table was then imported into R for generating bar plots. ClueGO v2.5.7 (36) add-on of the Cytoscape Platform v3.72 (37) was used to generate **Figures 1C, D**. For **Figure 5A**, a table containing official names and corresponding foldchange values of the 80 genes that are common across all three datasets was used as input for the Ingenuity Pathway Analysis (IPA) software (Qiagen). The core analysis function of the software was used to interpret the data and generate the graph (**Figure 5A**) of canonical pathways that correspond to the changes in gene expression. **Figures 5B–D** were generated from the genes enriched in the indicated canonical pathways using STRING v11 (38–41), and the clusters were defined based on likelihood of protein-protein interaction using the STRING’s implementation of the Markov cluster algorithm (MCL) clustering (42). Sequencing data generated for this study has been deposited in the Gene Expression Omnibus (GEO) database under the accession number GSE157159.

### Determination of Immune Cell Contribution to Gene Expression

Transcripts per million (TPM) normalized gene expression matrix was generated according to the method proposed by Wagner *et al. (* 43) and was used as input for estimating cell type enrichment using the xCell (44) program with default settings. The resulting cell type estimation matrix was filtered to remove cell types with enrichment scores less than 0.1 and used as input for generating the heatmap in **Figure 4**.

### Protein Expression Dataset

Differentially expressed genes (DEGs) with absolute foldchange values greater than 1 between pSS and non-SS control subjects were separated into upregulated and downregulated genes. Each subset was then used to filter the RNA gene expression values from tissues downloaded from the Human Protein Atlas (HPA) (45). The salivary gland and several immune-related tissues were then further extracted and used to generate the boxplots visualizing the estimated contribution of these tissues to the overall differential gene expression profiles observed between the non-SS control and pSS tissues.

## Results

### Defining the Transcriptome of the Minor Salivary Gland of pSS Patients

To better define the global gene expression patterns of pSS and to identify new molecular players that may contribute to the development of this disease, we performed RNA-sequencing based expression profiling of human MSGs from eight non-SS controls and nine pSS patients. In order to better analyze the overall changes in gene expression patterns between cases and controls, we utilized principal component analysis (PCA). The resulting PCA plot revealed a clear degree of separation between the different samples with each of the control and pSS patient groups appearing as separate entities (**Figure 1A**). Overall, our analysis suggests that the patient samples segregated well based on their mRNA expression profiles.

To better appreciate the underlying molecular drivers of pSS, we compared the transcriptomic profiles of non-SS control and pSS MSGs. Our analysis identified 5529 differentially expressed genes (DEGs), with 2979 genes upregulated and 2550 genes downregulated with the top 100 upregulated and downregulated genes shown in **Figure 1B** (**Figure 1B** and **Supplementary Table S2**). To better appreciate the biological relevance of the global transcriptomic differences between the control and pSS glands, we performed pathway analysis based on the top 100 DEGs. As expected, in the glands of pSS patients we observed specific enrichment of biological processes associated with immune responses including B cell proliferation, regulatory T cell differentiation, regulation of interleukin-12 production and chemokine-mediated signaling pathway—many of which have been implicated in SS pathogenesis (17, 46, 47) (**Figure 1C** and **Supplementary Table S3**). In contrast, downregulated genes were associated with negative regulation of cell-cell adhesion, O-glycan processing, and glycoprotein metabolic process, all of which are important processes necessary for proper salivary gland function including host-mediated defense mechanisms (**Figure 1D** and **Supplementary Table S4**) (48).

### Integrated Analysis Identifies a pSS Molecular Signature

**Figure 2:**
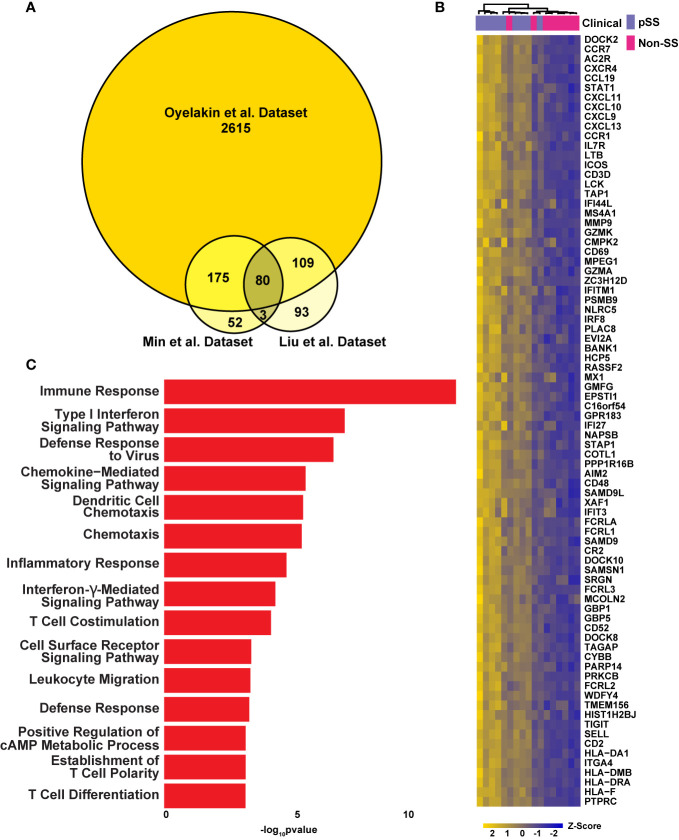
Integrated Analysis Reveals a pSS Molecular Signature. **(A)** Venn diagram displaying the overlay of up-regulated genes identified in the RNA-seq dataset described here (Oyelakin et al.) and Min et al. (50) and Liu et al. (49) datasets. **(B)** Heatmap visualization of the 80 common upregulated genes identified in panel A. **(C)** Bar plot highlights biological processes enriched in the 80 common upregulated genes identified in panel A above.

To confirm the robustness of our sequencing results, we next compared the global transcriptomes of the control and pSS glands by utilizing RNA-sequencing (RNA-seq) datasets described here, to additional RNA-seq (Liu et al.) (49) and microarray array datasets (Min et al.) (50) that have been previously reported (**Figure 2** and **Supplementary Figure S1**). Given that our analysis revealed a significant number of upregulated DEGs in our dataset (Oyelakin et al.) compared to the Liu et al. and Min et al. datasets, we focused on the 80 upregulated DEGs that were enriched across each of the three datasets (**Figures 2A, B** and **Supplementary Table S2**). We reasoned that this approach would address possible variations between the three datasets due to technical and experimental differences. Indeed, our analysis identified a number of genes common to all three datasets that have been previously demonstrated to play important roles in pSS pathogenesis including several members of the CXC chemokine family of cytokines including *CXCL9, CXCL10, CXCL11, CXCL13* (15, 51, 52), members of the guanylate-binding protein family; *GBP1*, *GBP5* (49, 53, 54), as well as *AIM2* (55), *CD52* (56), and *GZMK* (57). To further confirm these results, we performed quantitative reverse-transcription polymerase chain reaction (qRT-PCR) using a subset of patient samples which were included in our RNA-seq analysis to examine the mRNA expression levels of a select number of candidate genes, several of which have been implicated in pSS pathogenesis. Concordant with our RNA-seq results, we found reduced mRNA expression levels of *INSIG1* (19) and elevated expression levels of *CCL5*, *LYN*, *STAT1*, *IL7R*, and *IRF1*, all of which have been previously implicated in pSS (50, 57–61) (**Supplementary Figure S2**). In order to further validate these findings, we performed additional qRT-PCR analyses using an independent cohort of four pairs of pSS and control patient samples and observed similar trends in overall gene expression (**Supplementary Figure S3** and **Supplementary Table S5**). Having identified 80 common genes across the three datasets, we next sought to gain a better understanding of the underlying biological functions and pathways associated with these genes. Not surprisingly, our analysis identified enrichment of genes associated with a number of biological processes which have been previously linked to pSS including immune responses, type I interferon signaling pathway, defense response to virus, chemokine-mediated signaling pathway, inflammatory response, and interferon-γ-mediated signaling pathway (17, 18, 62, 63) (**Figure 2C** and **Supplementary Table S6**). Taken together, our integrated analysis has identified a molecular signature which includes a number of genes and pathways which have previously been shown to play a role in pSS, highlighting the power of this analysis.

### Defining the Nature of the Transcriptomic Changes in pSS Salivary Glands

**Figure 3:**
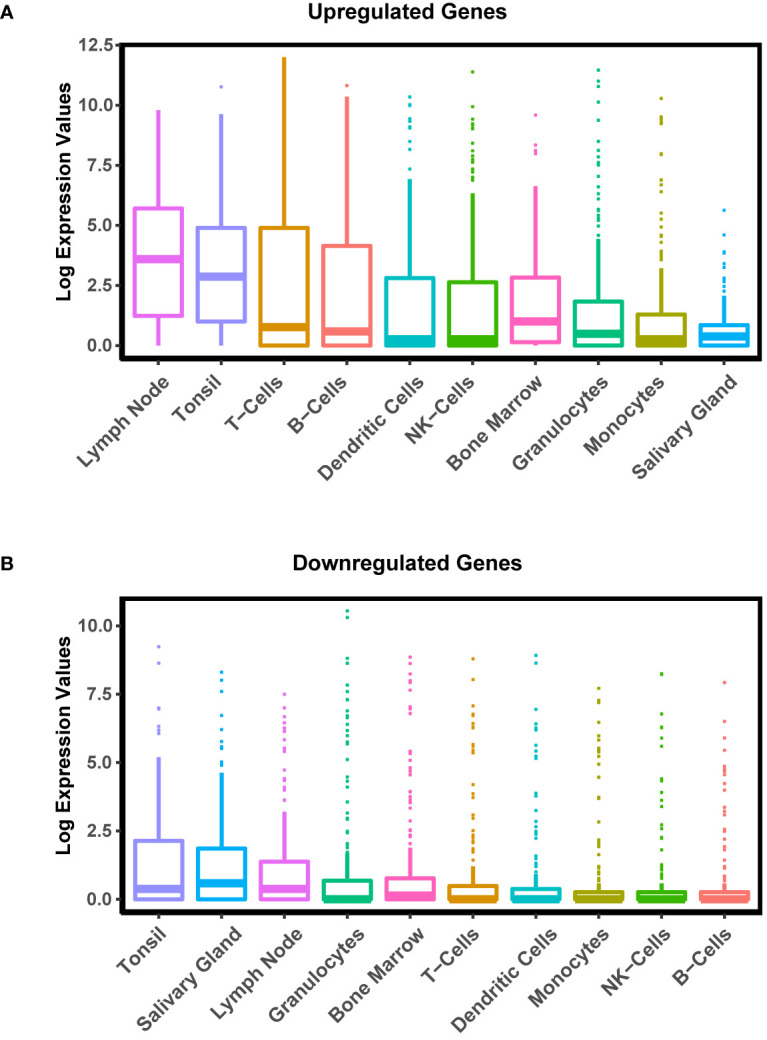
Cell and Tissue Specific Distribution of Differentially Expressed Genes in pSS. Boxplot displaying the distribution of expression of upregulated **(A)** and downregulated **(B)** genes in pSS patient glands in various tissues and cell types generated by the Human Protein Atlas (HPA) project.

To further evaluate the changes in gene expression in pSS and better define the overall nature of these transcriptomic changes, we mined the publicly available transcriptome database for human tissues generated by the HPA project (45). Upon comparison of the average gene expression levels of the pSS salivary gland datasets to various human tissue datasets, we found select enrichment of genes in the pSS patient samples to be those that are predominantly expressed in immune-specific tissues and cell types (**Figure 3A**). Given this result, it is tempting to speculate that the specific enrichment of genes associated with immune organs and tissues is a reflection of immune cell infiltration commonly observed in the glands of pSS patients. Conversely, we found the genes that were downregulated in pSS patients were highly enriched in the tonsil and salivary gland tissues compared to other immune tissues (**Figure 3B**). This suggests that in addition to the immune cell-type related changes, other salivary gland specific cell types such as epithelial cells, may also be affected in pSS (64, 65).

**Figure 4:**
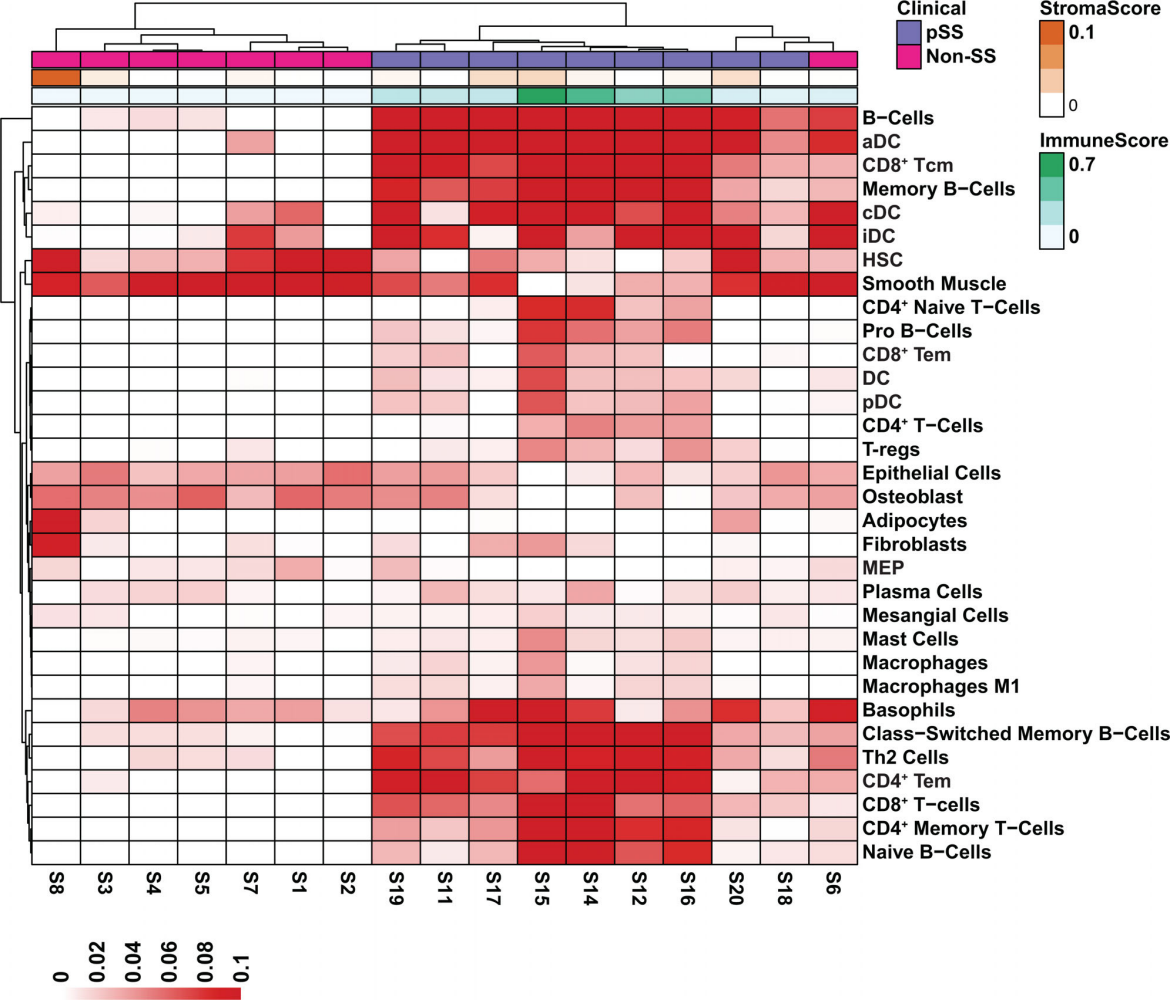
Cell Type Enrichment Analysis in pSS. Heatmap visualization of gene set enrichment scores by cell type. Scores were normalized across rows. The stroma and immune scores are displayed at the top of the heatmap. Cell types were assigned by the xCell algorithm based on the estimated enrichment of cell type signature genes in each bulk RNA-seq sample.

Armed with a global view of the overall transcriptional changes in the glands of pSS patients, we next sought to characterize the cell types that may be contributing to the alterations in gene expression. Towards this end we utilized xCell (44), a cell type enrichment analysis tool that allowed us to estimate the cell types contributing to the bulk RNA-seq expression profile using published information from single-cell RNA-seq datasets (66–68). As expected, we observed enrichment of genes associated with various immune cells types including B cells, conventional dendritic cells (cDC), plasmacytoid dendritic cells (pDC) and CD4^+^ effector memory T cells (CD4^+^ Tem) in the pSS glands, which is in good agreement with previous reports (69–74) (**Figure 4**). Interestingly, our analysis demonstrated a decrease in genes commonly expressed in epithelial cells (**Figure 4**). Indeed, these results correlate well with our own DEG analysis demonstrating that genes which were downregulated in pSS were highly enriched in normal salivary gland tissues as described in **Figure 3B**. Taken together, our results highlight the complex cell intrinsic and extrinsic changes occurring in the glands of pSS patients.

### Functional Gene Regulatory Network Analysis

**Figure 5:**
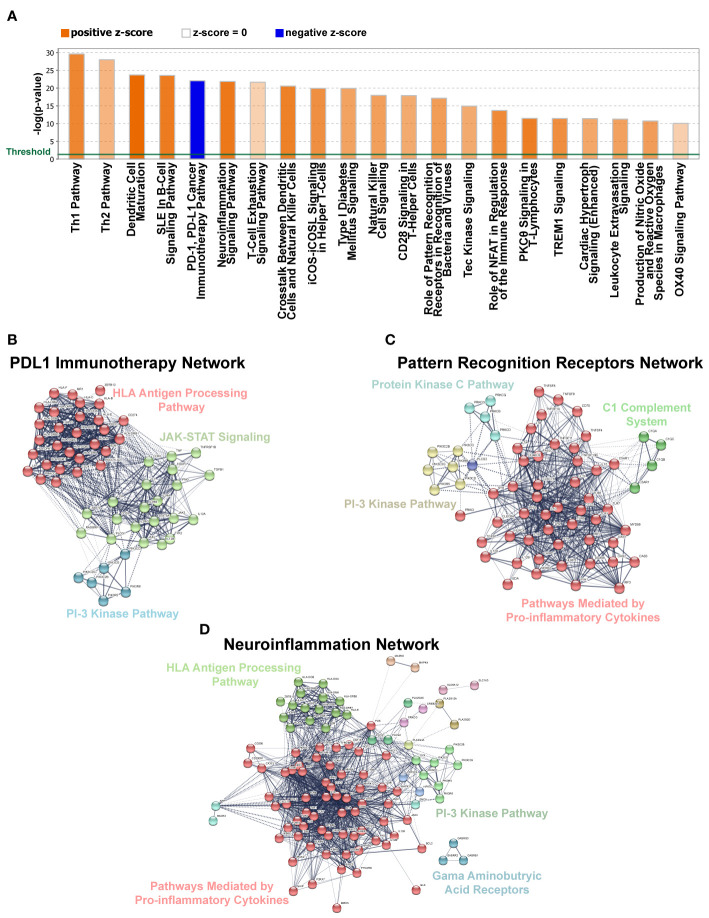
Gene Networks and Canonical Pathways Generated by Ingenuity Pathway Analysis (IPA). **(A)** Top 21 significantly affected canonical pathways for DEGs identified in our dataset based on IPA. Bars denote the different pathways based on Z-scores (2.0 < Z score < −2.0). Orange color indicates pathway activation, while blue denotes suppression. **(B–D)** Gene network analysis using IPA for pathways selected from panel A above. Solid lines denote high correlation between members of a defined cluster and dotted lines denote weak inter-cluster correlation.

Over the years, gene regulatory network analyses have emerged as an important tool in identifying transcriptional control programs, regulatory relationships and signaling networks that operate in the gene-rich environments during development and in disease. Towards this end, we utilized ingenuity pathway analysis (IPA) to explore differences in pathways and gene regulatory networks between the pSS and non-pSS control samples. Our analysis revealed the top two differentially regulated pathways to include the T-helper cell pathways—Th1 and Th2 (**Figure 5A**), which is in good agreement with previous studies detailing the role of T cells in SS (75). Surprisingly, we also observed specific enrichment of DEGs that are commonly associated with repression of the PD-1/PDL-1 cancer immunotherapy pathway (**Figure 5A** and **Supplementary Table S7**). The possible association of pSS with repressed PD-1/PDL-1 pathway is surprising given that some studies have reported elevated expression levels of PD-1 and PDL-1 in the salivary glands of pSS patients (76–78). However, our finding is in agreement with recent reports of the development of a Sjögren’s like syndrome in cancer patients treated with PD-1/PD-L1 checkpoint inhibitors (79, 80). Further support for pSS development resulting from repressed PD-1/PDL-1 comes from mouse models which have demonstrated that animals with deletion of PD-1 develop autoimmune diseases that include lupus-like arthritis and glomerulonephritis (81, 82). Given the conflicting roles for the PD-1/PDL-1 pathway in pSS pathogenesis, further careful studies are warranted. Yet another interesting finding was the observed enrichment of genes associated with the neuroinflammation signaling pathway (**Figure 5A**). This is in line with recent studies examining the role of the neuroendocrine system in systemic autoimmune diseases including rheumatoid arthritis, SLE and SS (83, 84). Finally, the role of pattern recognition receptors in recognition of bacteria and viruses was also among the over-represented pathways in our DEG dataset, which is in accordance with emerging evidence, suggesting a role for toll-like receptor (TLR) activation in pSS (**Figure 5A**) (85). Gene regulatory network analyses detailing the signaling networks connecting the DEGs for each of the aforementioned pathways are demonstrated in **Figures 5B–D**. Overall, while our RNA-seq driven data analysis revealed enrichment in a number of immune-mediated functional categories, it has also provided clues to the involvement of other pathways in pSS, some of which have only recently received attention.

## Discussion

While the underlying molecular mechanisms driving the pathogenesis of SS has been an area of extensive research over the last several decades, very few advancements have been made in treating this disease. Although various strategies have been employed over the years to address this, recent advances in next-generation sequencing approaches like RNA-seq have provided unprecedented insight into the complex molecular circuitry that governs dysregulated transcriptional networks contributing to diseased states. Here we have utilized RNA-seq to generate a comprehensive global gene expression profile of MSGs from patients with pSS. Using an integrative based approach, we have identified a pSS molecular gene signature that may offer insight into new players and pathways important for driving this disease. Moreover, we have utilized sophisticated computational and bioinformatics-driven analyses to identify important cell types that may be contributing to the underlying transcriptomic changes in the MSGs of pSS patients.

Our RNA-seq based approach identified a large number (~5529) of DEGs between non-SS control and pSS glands. More specifically, our analysis revealed 2979 upregulated and 2550 downregulated genes. These results were particularly surprising given that a recent RNA-seq study performed by Liu et al., and an integrated microarray-based study by Min et al., revealed a total of 293 and 382 DEGs, respectively (49, 50). While discrepancies between the number of DEGs identified in our study detailed here, compared to the microarray-based study can be attributed to the inherent limitations associated with microarray technologies, the differences we observed between our study and that of the RNA-seq study performed by Liu et al., is particularly intriguing. A potential explanation for this discrepancy could be ascribed, among many things, to differences in patient background and demographics, disease severity, focus score and the inherent variability associated with genomic data collection and processing. We suspect that the patients included in the Liu et al. study were all of Chinese descent and thus likely represent a more homogenous patient population. Conversely, the patients included in our analysis were more ethnically diverse (**Table 1**). Hence, it is tempting to speculate that the increased number of DEGs observed in our data set is a direct reflection of ethnic diversity and more representative of the genetic heterogeneity commonly associated with this disease (86, 87). It is also plausible that the differences in focus scores between the two patient groups may in part account for the significant difference in DEGs. Indeed, the high average patient focus scores (8.4) of our study compared to that of Liu et al. (1.8), might be indicative of enhanced immune cell activation in the minor salivary gland biopsies chosen for our study. We suspect that this heightened immune activation might broadly influence not only the inflammatory infiltrates but also the epithelial cell populations, which may reflect the differences in DEGs. Future studies aimed at more detailed investigations of the dynamic changes in the transcriptomic landscape as it pertains to patient focus scores will be of both prognostic and therapeutic value.

One important innovative aspect of our study is the generation of a pSS molecular signature which we posit includes relevant genes, genetic biomarkers and pathways—this can be extremely revealing about the underlying mechanisms driving pSS pathogenesis. While our molecular signature has identified a number of genes which have previously been reported to play key roles in pSS, we have also uncovered several genes which have not been previously associated with this disease and which may serve as candidates for future studies. For instance, we identified the T cell activation GTPase-activating protein (*TAGAP*) gene as an integral component of the pSS molecular gene signature. Although *TAGAP* has not been directly linked to SS, prior GWAS studies have identified single nucleotide polymorphisms (SNPs) located within the TAGAP genomic locus to be genetic risk factors for rheumatoid arthritis, a common autoimmune disease (88). Additionally, we identified the Mucolipin 2 (*MCOLN2*) gene to be part of our gene signature. The Mucolipin 2 gene encodes for the transient receptor potential mucolipin channel 2 (TRPML2) protein which belongs to the TRPML family of cation channel proteins (89). Recent studies have suggested a role for *MCOLN2* in the activation of innate immune responses through TLR signaling (90). This is an interesting correlation given the emerging role for TLR signaling in pSS pathobiology (85, 91–93).

While glandular destruction observed in SS patients has commonly been attributed to the consequences of abnormal B cell and T cell responses, emerging evidence also suggests a pathogenic role for epithelial cells in contributing to disease development and progression. Indeed, salivary gland epithelial cells (SGECs) isolated from patients with SS have been shown to play an active role in driving local autoimmune responses by mediating recruitment, homing, activation and differentiation of immune cells (94–96). In fact, our computational analyses clearly demonstrated alterations to the transcriptional landscape of the epithelial cells in pSS patients, lending further credence to an active role for this cell type in disease pathology. Although our bulk RNA-seq studies described here have been valuable in examining global changes of protein-coding genes, other genomic approaches focusing on the roles of microRNAs (miRNA) and circular RNAs (circRNAs) have also been gaining interest and have been revelatory for pSS biology (97, 98). Additional follow-up RNA-seq studies with larger cohorts of patients and more importantly, single-cell based studies that can delve deeper into examining alterations in the molecular and cellular heterogeneity of the various cell types of the MSGs of SS patients will be extremely valuable. As the genomics era continues to advance and evolve, it will no doubt shed new light on the underlying mechanisms driving SS with the hope of offering new avenues to treat this disease.

## Data Availability Statement

The datasets presented in this study can be found in online repositories. The names of the repository/repositories and accession number(s) can be found below: https://www.ncbi.nlm.nih.gov/geo/, GSE157159.

## Ethics Statement

The studies involving human participants were reviewed and approved by the Oklahoma Medical Research Foundation and the Augusta University Dental College of Georgia, Institutional Review Boards. The patients/participants provided their written informed consent to participate in this study.

## Author Contributions

AO and R-AR designed the experiments. AO, EH, ES, SM, MC, SS, JK, and R-AR performed experiments. AO, EH, and R-AR analyzed the data. CL, AR, LR, RS, DL, DS, KG, KS, SD, ZK, and AF provided essential tools. AO and R-AR wrote the paper. All authors contributed to the article and approved the submitted version.

## Funding

This work was supported by grants AR060804 (KS, RS, AF, and CL) and AR065953 (CL) from the National Institutes of Health/National Institute of Arthritis and Musculoskeletal and Skin Diseases (NIH/NIAMS). AO, EH, ES, and SM were supported by the State University of New York at Buffalo, School of Dental Medicine, Department of Oral Biology training grant from the NIH/National Institute of Dental and Craniofacial Research (NIH/NIDCR) DE023526. The content is solely the responsibility of the authors and does not necessarily represent the official views of the National Institutes of Health.

## Conflict of Interest

The authors declare that the research was conducted in the absence of any commercial or financial relationships that could be construed as a potential conflict of interest.

## References

[1] Brito-Zeron P Baldini C Bootsma H Bowman SJ Jonsson R Mariette X Sjogren syndrome. Nat Rev Dis Primers (2016) 2:16047. 10.1038/nrdp.2016.47 27383445

[2] Maciel G Crowson CS Matteson EL Cornec D Prevalence of Primary Sjogren’s Syndrome in a US Population-Based Cohort. Arthritis Care Res (Hoboken) (2017) 69(10):1612–6. 10.1002/acr.23173 PMC5478481 27998024

[3] Zhang L Xu P Wang X Zhang Z Zhao W Li Z Identification of differentially expressed genes in primary Sjogren’s syndrome. J Cell Biochem (2019) 120(10):17368–77. 10.1002/jcb.29001 31125139

[4] Flores-Chavez A Kostov B Solans R Fraile G Maure B Feijoo-Masso C Severe, life-threatening phenotype of primary Sjogren’s syndrome: clinical characterisation and outcomes in 1580 patients (GEAS-SS Registry). Clin Exp Rheumatol (2018) 36 Suppl 112(3):121–9. 30156546

[5] Delaleu N Jonsson R Koller MM Sjogren’s syndrome. Eur J Oral Sci (2005) 113(2):101–13. 10.1111/j.1600-0722.2004.00183.x 15819815

[6] Gottenberg JE Busson M Loiseau P Cohen-Solal J Lepage V Charron D In primary Sjogren’s syndrome, HLA class II is associated exclusively with autoantibody production and spreading of the autoimmune response. Arthritis Rheum (2003) 48(8):2240–5. 10.1002/art.11103 12905478

[7] Fox RI Pearson G Vaughan JH Detection of Epstein-Barr virus-associated antigens and DNA in salivary gland biopsies from patients with Sjogren’s syndrome. J Immunol (1986) 137(10):3162–8. 3021847

[8] Jorgensen C Legouffe MC Perney P Coste J Tissot B Segarra C Sicca syndrome associated with hepatitis C virus infection. Arthritis Rheum (1996) 39(7):1166–71. 10.1002/art.1780390714 8670326

[9] Quaresma JA Yoshikawa GT Koyama RV Dias GA Fujihara S Fuzii HT HTLV-1, Immune Response and Autoimmunity. Viruses (2015) 8(1). 10.3390/v8010005 PMC4728565 26712781

[10] Mofors J Arkema EV Bjork A Westermark L Kvarnstrom M Forsblad-d’Elia H Infections increase the risk of developing Sjogren’s syndrome. J Intern Med (2019) 285(6):670–80. 10.1111/joim.12888 30892751

[11] Weller ML Gardener MR Bogus ZC Smith MA Astorri E Michael DG Hepatitis Delta Virus Detected in Salivary Glands of Sjogren’s Syndrome Patients and Recapitulates a Sjogren’s Syndrome-Like Phenotype in Vivo. Pathog Immun (2016) 1(1):12–40. 10.20411/pai.v1i1.72 27294212 PMC4902173

[12] Youinou P Sjogren’s syndrome: a quintessential B cell-induced autoimmune disease. Joint Bone Spine (2008) 75(1):1–2. 10.1016/j.jbspin.2007.07.001 17905630

[13] Hansen A Gosemann M Pruss A Reiter K Ruzickova S Lipsky PE Abnormalities in peripheral B cell memory of patients with primary Sjogren’s syndrome. Arthritis Rheum (2004) 50(6):1897–908. 10.1002/art.20276 15188366

[14] Christodoulou MI Kapsogeorgou EK Moutsopoulos NM Moutsopoulos HM Foxp3+ T-regulatory cells in Sjogren’s syndrome: correlation with the grade of the autoimmune lesion and certain adverse prognostic factors. Am J Pathol (2008) 173(5):1389–96. 10.2353/ajpath.2008.080246 PMC2570129 18818377

[15] Kramer JM Klimatcheva E Rothstein TL CXCL13 is elevated in Sjogren’s syndrome in mice and humans and is implicated in disease pathogenesis. J Leukoc Biol (2013) 94(5):1079–89. 10.1189/jlb.0113036 PMC3800060 23904442

[16] Barone F Bombardieri M Manzo A Blades MC Morgan PR Challacombe SJ Association of CXCL13 and CCL21 expression with the progressive organization of lymphoid-like structures in Sjogren’s syndrome. Arthritis Rheum (2005) 52(6):1773–84. 10.1002/art.21062 15934082

[17] Blokland SLM Flessa CM van Roon JAG Mavragani CP Emerging roles for chemokines and cytokines as orchestrators of immunopathology in Sjogren’s syndrome. Rheumatol (Oxford) (2019). 10.1093/rheumatology/key438 30838419

[18] Li H Ice JA Lessard CJ Sivils KL Interferons in Sjogren’s Syndrome: Genes, Mechanisms, and Effects. Front Immunol (2013) 4:290. 10.3389/fimmu.2013.00290 24062752 PMC3778845

[19] Gottenberg JE Cagnard N Lucchesi C Letourneur F Mistou S Lazure T Activation of IFN pathways and plasmacytoid dendritic cell recruitment in target organs of primary Sjogren’s syndrome. Proc Natl Acad Sci U S A (2006) 103(8):2770–5. 10.1073/pnas.0510837103 PMC1413808 16477017

[20] Leehan KM Pezant NP Rasmussen A Grundahl K Moore JS Radfar L Fatty infiltration of the minor salivary glands is a selective feature of aging but not Sjogren’s syndrome. Autoimmunity (2017) 50(8):451–7. 10.1080/08916934.2017.1385776 PMC5730459 28988489

[21] Vitali C Bombardieri S Jonsson R Moutsopoulos HM Alexander EL Carsons SE Classification criteria for Sjogren’s syndrome: a revised version of the European criteria proposed by the American-European Consensus Group. Ann Rheum Dis (2002) 61(6):554–8. 10.1136/ard.61.6.554 PMC1754137 12006334

[22] Shiboski CH Shiboski SC Seror R Criswell LA Labetoulle M Lietman TM 2016 American College of Rheumatology/European League Against Rheumatism classification criteria for primary Sjogren’s syndrome: A consensus and data-driven methodology involving three international patient cohorts. Ann Rheum Dis (2017) 76(1):9–16. 10.1136/annrheumdis-2016-210571 27789466

[23] Shiboski CH Shiboski SC Seror R Criswell LA Labetoulle M Lietman TM 2016 American College of Rheumatology/European League Against Rheumatism Classification Criteria for Primary Sjogren’s Syndrome: A Consensus and Data-Driven Methodology Involving Three International Patient Cohorts. Arthritis Rheumatol (2017) 69(1):35–45. 10.1136/annrheumdis-2016-210571 27785888 PMC5650478

[24] Daniels TE Cox D Shiboski CH Schiodt M Wu A Lanfranchi H Associations between salivary gland histopathologic diagnoses and phenotypic features of Sjogren’s syndrome among 1,726 registry participants. Arthritis Rheumatol (2011) 63(7):2021–30. 10.1002/art.30381 PMC3128201 21480190

[25] Kim D Paggi JM Park C Bennett C Salzberg SL Graph-based genome alignment and genotyping with HISAT2 and HISAT-genotype. Nat Biotechnol (2019) 37(8):907–15. 10.1038/s41587-019-0201-4 PMC7605509 31375807

[26] Langmead B Salzberg SL Fast gapped-read alignment with Bowtie 2. Nat Methods (2012) 9(4):357–9. 10.1038/nmeth.1923 PMC3322381 22388286

[27] Liao Y Smyth GK Shi W featureCounts: an efficient general purpose program for assigning sequence reads to genomic features. Bioinformatics (2014) 30(7):923–30. 10.1093/bioinformatics/btt656 24227677

[28] Rahman M Jackson LK Johnson WE Li DY Bild AH Piccolo SR Alternative preprocessing of RNA-Sequencing data in The Cancer Genome Atlas leads to improved analysis results. Bioinformatics (2015) 31(22):3666–72. 10.1093/bioinformatics/btv377 PMC4804769 26209429

[29] Love MI Huber W Anders S Moderated estimation of fold change and dispersion for RNA-seq data with DESeq2. Genome Biol (2014) 15(12):550. 10.1186/s13059-014-0550-8 25516281 PMC4302049

[30] Huang DW Sherman BT Tan Q Collins JR Alvord WG Roayaei J The DAVID Gene Functional Classification Tool: a novel biological module-centric algorithm to functionally analyze large gene lists. Genome Biol (2007) 8(9):R183. 10.1186/gb-2007-8-9-r183 17784955 PMC2375021

[31] Sherman BT Huang da W Tan Q Guo Y Bour S Liu D DAVID Knowledgebase: a gene-centered database integrating heterogeneous gene annotation resources to facilitate high-throughput gene functional analysis. BMC Bioinf (2007) 8:426. 10.1186/1471-2105-8-426 PMC2186358 17980028

[32] Du J Yuan Z Ma Z Song J Xie X Chen Y KEGG-PATH: Kyoto encyclopedia of genes and genomes-based pathway analysis using a path analysis model. Mol Biosyst (2014) 10(9):2441–7. 10.1039/C4MB00287C 24994036

[33] Kanehisa M Goto S KEGG: kyoto encyclopedia of genes and genomes. Nucleic Acids Res (2000) 28(1):27–30. 10.1093/nar/28.1.27 10592173 PMC102409

[34] Ogata H Goto S Sato K Fujibuchi W Bono H Kanehisa M KEGG: Kyoto Encyclopedia of Genes and Genomes. Nucleic Acids Res (1999) 27(1):29–34. 10.1093/nar/27.1.29 9847135 PMC148090

[35] Wixon J Kell D The Kyoto encyclopedia of genes and genomes–KEGG. Yeast (2000) 17(1):48–55. 10.1002/(SICI)1097-0061(200004)17:1<48::AID-YEA2>3.0.CO;2-H 10928937 PMC2447041

[36] Bindea G Mlecnik B Hackl H Charoentong P Tosolini M Kirilovsky A ClueGO: a Cytoscape plug-in to decipher functionally grouped gene ontology and pathway annotation networks. Bioinformatics (2009) 25(8):1091–3. 10.1093/bioinformatics/btp101 PMC2666812 19237447

[37] Shannon P Markiel A Ozier O Baliga NS Wang JT Ramage D Cytoscape: a software environment for integrated models of biomolecular interaction networks. Genome Res (2003) 13(11):2498–504. 10.1101/gr.1239303 PMC403769 14597658

[38] von Mering C Huynen M Jaeggi D Schmidt S Bork P Snel B STRING: a database of predicted functional associations between proteins. Nucleic Acids Res (2003) 31(1):258–61. 10.1093/nar/gkg034 PMC165481 12519996

[39] Snel B Lehmann G Bork P Huynen MA STRING: a web-server to retrieve and display the repeatedly occurring neighbourhood of a gene. Nucleic Acids Res (2000) 28(18):3442–4. 10.1093/nar/28.18.3442 PMC110752 10982861

[40] von Mering C Jensen LJ Snel B Hooper SD Krupp M Foglierini M STRING: known and predicted protein-protein associations, integrated and transferred across organisms. Nucleic Acids Res (2005) 33(Database issue):D433–7. 10.1093/nar/gki005 PMC539959 15608232

[41] Szklarczyk D Gable AL Lyon D Junge A Wyder S Huerta-Cepas J STRING v11: protein-protein association networks with increased coverage, supporting functional discovery in genome-wide experimental datasets. Nucleic Acids Res (2019) 47(D1):D607–D13. 10.1093/nar/gky1131 PMC6323986 30476243

[42] Enright AJ Van Dongen S Ouzounis CA An efficient algorithm for large-scale detection of protein families. Nucleic Acids Res (2002) 30(7):1575–84. 10.1093/nar/30.7.1575 PMC101833 11917018

[43] Wagner GP Kin K Lynch VJ Measurement of mRNA abundance using RNA-seq data: RPKM measure is inconsistent among samples. Theory Biosci (2012) 131(4):281–5. 10.1007/s12064-012-0162-3 22872506

[44] Aran D Hu Z Butte AJ xCell: digitally portraying the tissue cellular heterogeneity landscape. Genome Biol (2017) 18(1):220. 10.1186/s13059-017-1349-1 29141660 PMC5688663

[45] Uhlen M Fagerberg L Hallstrom BM Lindskog C Oksvold P Mardinoglu A Proteomics. Tissue-based map of the human proteome. Science (2015) 347(6220):1260419. 10.1126/science.1260419 25613900

[46] Qi J Li D Shi G Zhang X Pan Y Dou H Interleukin12 exacerbates Sjogren’s syndrome through induction of myeloidderived suppressor cells. Mol Med Rep (2019) 20(2):1131–8. 10.3892/mmr.2019.10352 PMC6625410 31173212

[47] Mavragani CP Moutsopoulos HM Sjogren’s syndrome. Annu Rev Pathol (2014) 9:273–85. 10.1146/annurev-pathol-012513-104728 24050623

[48] Cross BW Ruhl S Glycan recognition at the saliva - oral microbiome interface. Cell Immunol (2018) 333:19–33. 10.1016/j.cellimm.2018.08.008 30274839 PMC6296888

[49] Liu Z Li F Pan A Xue H Jiang S Zhu C Elevated CCL19/CCR7 Expression During the Disease Process of Primary Sjogren’s Syndrome. Front Immunol (2019) 10:795. 10.3389/fimmu.2019.00795 31068931 PMC6491632

[50] Min HK Moon SJ Park KS Kim KJ Integrated systems analysis of salivary gland transcriptomics reveals key molecular networks in Sjogren’s syndrome. Arthritis Res Ther (2019) 21(1):294. 10.1186/s13075-019-2082-9 31856901 PMC6921432

[51] Ogawa N Ping L Zhenjun L Takada Y Sugai S Involvement of the interferon-gamma-induced T cell-attracting chemokines, interferon-gamma-inducible 10-kd protein (CXCL10) and monokine induced by interferon-gamma (CXCL9), in the salivary gland lesions of patients with Sjogren’s syndrome. Arthritis Rheumatol (2002) 46(10):2730–41. 10.1002/art.10577 12384933

[52] Ogawa N Kawanami T Shimoyama K Ping L Sugai S Expression of interferon-inducible T cell alpha chemoattractant (CXCL11) in the salivary glands of patients with Sjogren’s syndrome. Clin Immunol (2004) 112(3):235–8. 10.1016/j.clim.2004.03.008 15308116

[53] Imgenberg-Kreuz J Sandling JK Bjork A Nordlund J Kvarnstrom M Eloranta ML Transcription profiling of peripheral B cells in antibody-positive primary Sjogren’s syndrome reveals upregulated expression of CX3CR1 and a type I and type II interferon signature. Scand J Immunol (2018) 87(5):e12662. 10.1111/sji.12662 29655283

[54] Nezos A Gravani F Tassidou A Kapsogeorgou EK Voulgarelis M Koutsilieris M and II interferon signatures in Sjogren’s syndrome pathogenesis: Contributions in distinct clinical phenotypes and Sjogren’s related lymphomagenesis. J Autoimmun (2015) 63:47–58. 10.1016/j.jaut.2015.07.002 26183766 PMC4564326

[55] Vakrakou AG Svolaki IP Evangelou K Gorgoulis VG Manoussakis MN Cell-autonomous epithelial activation of AIM2 (absent in melanoma-2) inflammasome by cytoplasmic DNA accumulations in primary Sjogren’s syndrome. J Autoimmun (2020) 108:102381. 10.1016/j.jaut.2019.102381 31919014

[56] Greenwell-Wild T Moutsopoulos NM Gliozzi M Kapsogeorgou E Rangel Z Munson PJ Chitinases in the salivary glands and circulation of patients with Sjogren’s syndrome: macrophage harbingers of disease severity. Arthritis Rheumatol (2011) 63(10):3103–15. 10.1002/art.30465 PMC3183169 21618203

[57] Hjelmervik TO Petersen K Jonassen I Jonsson R Bolstad AI Gene expression profiling of minor salivary glands clearly distinguishes primary Sjogren’s syndrome patients from healthy control subjects. Arthritis Rheumatol (2005) 52(5):1534–44. 10.1002/art.21006 15880807

[58] Pertovaara M Silvennoinen O Isomaki P Cytokine-induced STAT1 activation is increased in patients with primary Sjogren’s syndrome. Clin Immunol (2016) 165:60–7. 10.1016/j.clim.2016.03.010 26995659

[59] Bikker A Kruize AA Wenting M Versnel MA Bijlsma JW Lafeber FP Increased interleukin (IL)-7Ralpha expression in salivary glands of patients with primary Sjogren’s syndrome is restricted to T cells and correlates with IL-7 expression, lymphocyte numbers and activity. Ann Rheum Dis (2012) 71(6):1027–33. 10.1136/annrheumdis-2011-200744 22312161

[60] Zheng L Yu C Zhang Z Yang C Cai X Expression of interferon regulatory factor 1, 3, and 7 in primary Sjogren syndrome. Oral Surg Oral Med Oral Pathol Oral Radiol Endod (2009) 107(5):661–8. 10.1016/j.tripleo.2009.01.039 19426920

[61] Szodoray P Alex P Brun JG Centola M Jonsson R Circulating cytokines in primary Sjogren’s syndrome determined by a multiplex cytokine array system. Scand J Immunol (2004) 59(6):592–9. 10.1111/j.0300-9475.2004.01432.x 15182255

[62] Nguyen CQ Peck AB The Interferon-Signature of Sjogren’s Syndrome: How Unique Biomarkers Can Identify Underlying Inflammatory and Immunopathological Mechanisms of Specific Diseases. Front Immunol (2013) 4:142. 10.3389/fimmu.2013.00142 23847613 PMC3701867

[63] Kiripolsky J McCabe LG Kramer JM Innate immunity in Sjogren’s syndrome. Clin Immunol (2017) 182:4–13. 10.1016/j.clim.2017.04.003 28396235 PMC6025757

[64] Manoussakis MN Kapsogeorgou EK The role of epithelial cells in the pathogenesis of Sjogren’s syndrome. Clin Rev Allergy Immunol (2007) 32(3):225–30. 10.1007/s12016-007-8007-4 17992589

[65] Okuma A Hoshino K Ohba T Fukushi S Aiba S Akira S Enhanced apoptosis by disruption of the STAT3-IkappaB-zeta signaling pathway in epithelial cells induces Sjogren’s syndrome-like autoimmune disease. Immunity (2013) 38(3):450–60. 10.1016/j.immuni.2012.11.016 23453632

[66] Breschi A Munoz-Aguirre M Wucher V Davis CA Garrido-Martin D Djebali S A limited set of transcriptional programs define major cell types. Genome Res (2020) 30(7):1047–59. 10.1101/gr.263186.120 PMC7397875 32759341

[67] Bernard B Rajamanickam V Dubay C Piening B Alonso E Jutric Z Transcriptional and immunohistological assessment of immune infiltration in pancreatic cancer. PLoS One (2020) 15(8):e0238380. 10.1371/journal.pone.0238380 32866185 PMC7458344

[68] Pandey A Stawiski EW Durinck S Gowda H Goldstein LD Barbhuiya MA Integrated genomic analysis reveals mutated ELF3 as a potential gallbladder cancer vaccine candidate. Nat Commun (2020) 11(1):4225. 10.1038/s41467-020-17880-4 32839463 PMC7445288

[69] Nocturne G Mariette X B cells in the pathogenesis of primary Sjogren syndrome. Nat Rev Rheum (2018) 14(3):133–45. 10.1038/nrrheum.2018.1 29416129

[70] Lopes AP van Roon JAG Blokland SLM Wang M Chouri E Hartgring SAY MicroRNA-130a Contributes to Type-2 Classical DC-activation in Sjogren’s Syndrome by Targeting Mitogen- and Stress-Activated Protein Kinase-1. Front Immunol (2019) 10:1335. 10.3389/fimmu.2019.01335 31281310 PMC6595962

[71] Hillen MR Pandit A Blokland SLM Hartgring SAY Bekker CPJ van der Heijden EHM Plasmacytoid DCs From Patients With Sjogren’s Syndrome Are Transcriptionally Primed for Enhanced Pro-inflammatory Cytokine Production. Front Immunol (2019) 10:2096. 10.3389/fimmu.2019.02096 31552042 PMC6736989

[72] Ols ML Cullen JL Turqueti-Neves A Giles J Shlomchik MJ Dendritic Cells Regulate Extrafollicular Autoreactive B Cells via T Cells Expressing Fas and Fas Ligand. Immunity (2016) 45(5):1052–65. 10.1016/j.immuni.2016.10.005 PMC5112117 27793595

[73] Joachims ML Leehan KM Dozmorov MG Georgescu C Pan Z Lawrence C Sjogren’s Syndrome Minor Salivary Gland CD4(+) Memory T Cells Associate with Glandular Disease Features and have a Germinal Center T Follicular Helper Transcriptional Profile. J Clin Med (2020) 9(7). 10.3390/jcm9072164 PMC7408878 32650575

[74] Joachims ML Leehan KM Lawrence C Pelikan RC Moore JS Pan Z Single-cell analysis of glandular T cell receptors in Sjogren’s syndrome. JCI Insight (2016) 1(8). 10.1172/jci.insight.85609 PMC4922426 27358913

[75] Verstappen GM Kroese FGM Bootsma H T cells in primary Sjogren’s syndrome: targets for early intervention. Rheumatol (Oxford) (2019). 10.1093/rheumatology/kez004 PMC8516500 30770920

[76] Bolstad AI Eiken HG Rosenlund B Alarcon-Riquelme ME Jonsson R Increased salivary gland tissue expression of Fas, Fas ligand, cytotoxic T lymphocyte-associated antigen 4, and programmed cell death 1 in primary Sjogren’s syndrome. Arthritis Rheumatol (2003) 48(1):174–85. 10.1002/art.10734 12528117

[77] Kobayashi M Kawano S Hatachi S Kurimoto C Okazaki T Iwai Y Enhanced expression of programmed death-1 (PD-1)/PD-L1 in salivary glands of patients with Sjogren’s syndrome. J Rheum (2005) 32(11):2156–63. 16265694

[78] Li P Yang Y Jin Y Zhao R Dong C Zheng W B7-H3 participates in human salivary gland epithelial cells apoptosis through NF-kappaB pathway in primary Sjogren’s syndrome. J Transl Med (2019) 17(1):268. 10.1186/s12967-019-2017-x 31412888 PMC6694606

[79] Ramos-Casals M Maria A Suarez-Almazor ME Lambotte O Fisher BA Hernandez-Molina G Sicca/Sjogren’s syndrome triggered by PD-1/PD-L1 checkpoint inhibitors. Data from the International ImmunoCancer Registry (ICIR). Clin Exp Rheum (2019) 37 Suppl 118(3):114–22. 31464670

[80] Warner BM Baer AN Lipson EJ Allen C Hinrichs C Rajan A Sicca Syndrome Associated with Immune Checkpoint Inhibitor Therapy. Oncologist (2019) 24(9):1259–69. 10.1634/theoncologist.2018-0823 PMC6738284 30996010

[81] Nishimura H Nose M Hiai H Minato N Honjo T Development of lupus-like autoimmune diseases by disruption of the PD-1 gene encoding an ITIM motif-carrying immunoreceptor. Immunity (1999) 11(2):141–51. 10.1016/S1074-7613(00)80089-8 10485649

[82] Nishimura H Okazaki T Tanaka Y Nakatani K Hara M Matsumori A Autoimmune dilated cardiomyopathy in PD-1 receptor-deficient mice. Science (2001) 291(5502):319–22. 10.1126/science.291.5502.319 11209085

[83] Tzioufas AG Tsonis J Moutsopoulos HM Neuroendocrine dysfunction in Sjogren’s syndrome. Neuroimmunomodulation (2008) 15(1):37–45. 10.1159/000135622 18667798

[84] Johnson EO Skopouli FN Moutsopoulos HM Neuroendocrine manifestations in Sjogren’s syndrome. Rheum Dis Clin North Am (2000) 26(4):927–49. 10.1016/S0889-857X(05)70177-0 11084952

[85] Kiripolsky J Kramer JM Current and Emerging Evidence for Toll-Like Receptor Activation in Sjogren’s Syndrome. J Immunol Res (2018) 2018:1246818. 10.1155/2018/1246818 30671484 PMC6317121

[86] Imgenberg-Kreuz J Rasmussen A Sivils K Nordmark G Genetics and epigenetics in primary Sjogren’s syndrome. Rheumatol (Oxford) (2019). 10.1093/rheumatology/key330 PMC8121440 30770922

[87] Scofield RH Sharma R Pezant N Kelly JA Radfar L Lewis DM American Indians Have a Higher Risk of Sjogren’s Syndrome and More Disease Activity Than European Americans and African Americans. Arthritis Care Res (Hoboken) (2020) 72(8):1049–56. 10.1002/acr.24003 PMC6911033 31199565

[88] Chen R Stahl EA Kurreeman FA Gregersen PK Siminovitch KA Worthington J Fine mapping the TAGAP risk locus in rheumatoid arthritis. Genes Immun (2011) 12(4):314–8. 10.1038/gene.2011.8 PMC3114196 21390051

[89] Pedersen SF Owsianik G Nilius B TRP channels: an overview. Cell Calcium (2005) 38(3-4):233–52. 10.1016/j.ceca.2005.06.028 16098585

[90] Santoni G Morelli MB Amantini C Nabissi M Santoni M Santoni A Involvement of the TRPML Mucolipin Channels in Viral Infections and Anti-viral Innate Immune Responses. Front Immunol (2020) 11:739. 10.3389/fimmu.2020.00739 32425938 PMC7212413

[91] Kwok SK Cho ML Her YM Oh HJ Park MK Lee SY TLR2 ligation induces the production of IL-23/IL-17 via IL-6, STAT3 and NF-kB pathway in patients with primary Sjogren’s syndrome. Arthritis Res Ther (2012) 14(2):R64. 10.1186/ar3780 22417709 PMC3446432

[92] Ittah M Miceli-Richard C Gottenberg JE Sellam J Eid P Lebon P Viruses induce high expression of BAFF by salivary gland epithelial cells through TLR- and type-I IFN-dependent and -independent pathways. Eur J Immunol (2008) 38(4):1058–64. 10.1002/eji.200738013 18350548

[93] Spachidou MP Bourazopoulou E Maratheftis CI Kapsogeorgou EK Moutsopoulos HM Tzioufas AG Expression of functional Toll-like receptors by salivary gland epithelial cells: increased mRNA expression in cells derived from patients with primary Sjogren’s syndrome. Clin Exp Immunol (2007) 147(3):497–503. 10.1111/j.1365-2249.2006.03311.x 17302899 PMC1810489

[94] Tzioufas AG Kapsogeorgou EK Moutsopoulos HM Pathogenesis of Sjogren’s syndrome: what we know and what we should learn. J Autoimmun (2012) 39(1-2):4–8. 10.1016/j.jaut.2012.01.002 22326205

[95] Manoussakis MN Kapsogeorgou EK The role of intrinsic epithelial activation in the pathogenesis of Sjogren’s syndrome. J Autoimmun (2010) 35(3):219–24. 10.1016/j.jaut.2010.06.011 20685080

[96] Gong YZ Nititham J Taylor K Miceli-Richard C Sordet C Wachsmann D Differentiation of follicular helper T cells by salivary gland epithelial cells in primary Sjogren’s syndrome. J Autoimmun (2014) 51:57–66. 10.1016/j.jaut.2013.11.003 24411167

[97] Alevizos I Alexander S Turner RJ Illei GG MicroRNA expression profiles as biomarkers of minor salivary gland inflammation and dysfunction in Sjogren’s syndrome. Arthritis Rheum (2011) 63(2):535–44. 10.1002/art.30131 PMC3653295 21280008

[98] Li F Liu Z Zhang B Jiang S Wang Q Du L Circular RNA sequencing indicates circ-IQGAP2 and circ-ZC3H6 as noninvasive biomarkers of primary Sjogren’s syndrome. Rheumatol (Oxford) (2020). 10.1093/rheumatology/keaa163 32250392

