# Supplementary material for: Transcriptomic and Network Analysis of Minor Salivary Glands of Patients With Primary Sjögren’s Syndrome
Source: Front Immunol. 2021 Jan 8;11:606268. doi: 10.3389/fimmu.2020.606268 (PMC7821166; doi:10.3389/fimmu.2020.606268)
Supplement: Supplementary file 1 [file Table_1.docx]

**Supplemental Table S1. List of Primer Sequences for qRT-PCR.**

| Gene | Forward Primer (5' - 3') | Reverse Primer (5' - 3') |
| --- | --- | --- |
| CCL5 | CAG TCG TCT TTG TCA CCC GA | TTG ATG TAC TCC CGA ACC CA |
| IL7R | TAA TAG CTC AGG GGA GAT GGA | AAG ATG ACC AAC AGA GCG AC |
| INSIG1 | TGG CCT ACT GTA CCC CTG TA | TCC AAT TTA GCA CTG GCG TG |
| IRF1 | AAC TTC CAG GTG TCA CCC AT | TAG GTA CCC CTT CCC ATC CA |
| LYN | CGA GCG GGA AAT ATG GGA TG | TTG GAC GTT GGA TCT CTC AC |
| STAT1 | TCG ACA GTC TTG GCA CCT AA | CTG AGA CAT CCT GCC ACC TT |
| GAPDH | AGC CAC ATC GCT CAG ACA C | GCC CAA TAC GAC CAA ATC C |
