# Supplementary material for: Transcriptomic and Network Analysis of Minor Salivary Glands of Patients With Primary Sjögren’s Syndrome
Source: Front Immunol. 2021 Jan 8;11:606268. doi: 10.3389/fimmu.2020.606268 (PMC7821166; doi:10.3389/fimmu.2020.606268)
Supplement: Supplementary file 3 [file Table_3.docx]

| Supplementary Table S3 | |
| --- | --- |
| **Biological Processes** | **Associated Upregulated Genes** |
| B Cell Proliferation | CD19, CR2, CTLA4, FCRL3, IL21, MS4A1 |
| Regulation of B Cell Proliferation | CTLA4, FCRL3, IL21 |
| Regulation of Cell Killing | CD1A, CD1B, IL21, NCR1 |
| Positive Regulation of Cell Killing | CD1A, CD1B, IL21 |
| Regulation of Leukocyte Mediated Cytotoxicity | CD1A, CD1B, IL21, NCR1 |
| Positive Regulation of Leukocyte Mediated Cytotoxicity | CD1A, CD1B, IL21 |
| Response to Chemokine | CCL19, CCR7, CXCL10, CXCL11, CXCL13, CXCL9, CXCR3, CXCR5 |
| Cytokine Receptor Activity | CCR7, CXCR3, CXCR5, IL17REL, IL21R, IL22RA2, IL2RB |
| Chemokine-Mediated Signaling Pathway | CCL19, CCR7, CXCL10, CXCL11, CXCL13, CXCL9, CXCR3, CXCR5 |
| Cellular Response to Chemokine | CCL19, CCR7, CXCL10, CXCL11, CXCL13, CXCL9, CXCR3, CXCR5 |
| G Protein-Coupled Chemoattractant Receptor Activity | CCR7, CXCR3, CXCR5 |
| Chemokine Receptor Activity | CCR7, CXCR3, CXCR5 |
| C-C Chemokine Receptor Activity | CCR7, CXCR3, CXCR5 |
| Inflammatory Response to Antigenic Stimulus | CCR7, FUT7, PLA2G2D |
| Acute Inflammatory Response to Antigenic Stimulus | CCR7, FUT7, PLA2G2D |
| Regulation of Inflammatory Response to Antigenic Stimulus | CCR7, FUT7, PLA2G2D |
| Regulation of Acute Inflammatory Response | CCR7, FUT7, PLA2G2D |
| Regulation of Acute Inflammatory Response to Antigenic Stimulus | CCR7, FUT7, PLA2G2D |
| Regulation of Neutrophil Migration | CCL19, CCR7, FUT7 |
| Negative Regulation of T Cell Proliferation | CTLA4, IDO1, PLA2G2D |
| Regulatory T Cell Differentiation | CTLA4, FUT7, PLA2G2D |
| Interleukin-10 Production | IDO1, TIGIT, TNFRSF9 |
| Interleukin-12 Production | CCL19, CCR7, IDO1, LTB, TIGIT, TNFRSF9 |
| Regulation of Interleukin-10 Production | IDO1, TIGIT, TNFRSF9 |
| Regulation of Interleukin-12 Production | CCL19, CCR7, IDO1, LTB, TIGIT, TNFRSF9 |
| Negative Regulation of Interleukin-12 Production | CCR7, TIGIT, TNFRSF9 |
| Positive Regulation of Interleukin-12 Production | CCL19, CCR7, IDO1, LTB |
| Interleukin-12 Secretion | CCL19, CCR7, TNFRSF9 |
| Negative Regulation of Leukocyte Apoptotic Process | CCL19, CCR7, IDO1 |
| Regulation 3of Interleukin-12 Secretion | CCL19, CCR7, TNFRSF9 |
