# Supplementary material for: Transcriptomic and Network Analysis of Minor Salivary Glands of Patients With Primary Sjögren’s Syndrome
Source: Front Immunol. 2021 Jan 8;11:606268. doi: 10.3389/fimmu.2020.606268 (PMC7821166; doi:10.3389/fimmu.2020.606268)
Supplement: Supplementary file 4 [file Table_4.docx]

| Supplementary Table S4 | |
| --- | --- |
| **Biological Processes** | **Associated Downregulated Genes** |
| One-Carbon Metabolic Process | ALDH1L1, ALDH1L2, CA10, CA2, CA4, GNMT |
| Synapse Assembly | ADGRB3, ADGRL3, DRD2, ERBB4, FLRT3, NLGN1, NRG1, PCDHB10, PCDHB16, PCDHB3, SIX4, SLITRK3, SLITRK5, SLITRK6 |
| Organic Anion Transport | ATP8B1, CA2, CA4, CLTRN, CYB5R2, DBI, DRD2, IRS2, LRRC8E, NPY5R, NR4A2, PER2, PRKAA2, SLC16A14, SLC17A5, SLC26A2, SLC26A5, SLC2A10, SLC35A3, SLC38A4, SLC39A8, SLC66A1L, SLC6A15, TNFRSF11A, TRPC4 |
| Glycoprotein Metabolic Process | ALG14, B3GALT1, B3GNT3, B4GALT6, BACE2, CHST9, CNMD, COL11A1, GALNT12, GALNT3, GALNT5, GALNT7, GCNT3, GFPT1, HYAL1, MCFD2, MUC16, MUC21, NDNF, SLC2A10, SLC39A8 |
| O-Glycan Processing | B3GNT3, GALNT12, GALNT3, GALNT5, GALNT7, GCNT3, MUC16, MUC21 |
| Organic Acid Metabolic Process | ACADL, ACAT1, ADSS1, ALDH1A3, ALDH1L1, ALDH1L2, ALOX15B, APIP, ASRGL1, ATP8B1, B3GNT3, B4GALT6, BCKDHB, CHST6, CHST9, CRYM, CYP1A1, CYP2C19, CYP39A1, DCT, DDAH1, DDC, FA2H, GFPT1, GLYATL2, GNE, GNMT, GPT2, GSTA1, GSTO2, HOOK1, HYAL1, INSIG1, IRS2, ITIH6, IYD, MGST2, MIA3, MLXIPL, NDNF, OAZ3, OSBPL1A, PAM, PCDHB16, PER2, PRKAA2, PRKAR2B, PRUNE2, SLC26A2, SLC39A8, SULT2B1 |
| Oxoacid Metabolic Process | ACADL, ACAT1, ADSS1, ALDH1A3, ALDH1L1, ALDH1L2, ALOX15B, APIP, ASRGL1, ATP8B1, B3GNT3, B4GALT6, BCKDHB, CHST6, CHST9, CRYM, CYP1A1, CYP2C19, CYP39A1, DCT, DDAH1, DDC, FA2H, GFPT1, GLYATL2, GNE, GNMT, GPT2, GSTA1, GSTO2, HOOK1, HYAL1, INSIG1, IRS2, ITIH6, IYD, MGST2, MIA3, MLXIPL, NDNF, OAZ3, OSBPL1A, PAM, PCDHB16, PER2, PRKAA2, PRKAR2B, PRUNE2, SLC26A2, SLC39A8, SULT2B1 |
| Glutathione Derivative Metabolic Process | GSTA1, GSTA2, GSTO2, MGST1, MGST2 |
| Glutathione Derivative Biosynthetic Process | GSTA1, GSTA2, GSTO2, MGST1, MGST2 |
