# Supplementary material for: Transcriptomic and Network Analysis of Minor Salivary Glands of Patients With Primary Sjögren’s Syndrome
Source: Front Immunol. 2021 Jan 8;11:606268. doi: 10.3389/fimmu.2020.606268 (PMC7821166; doi:10.3389/fimmu.2020.606268)
Supplement: Supplementary file 5 [file Table_5.docx]

**Supplemental Table S5**

| Patient ID  (Sample ID) | Group | Age | Sex | Ethnicity | SSA | SSB | Focus Score |
| --- | --- | --- | --- | --- | --- | --- | --- |
| GRU-1306(S21) | Non-SS | 54 | F | White | - | - | 0 |
| GRU-1508(S22) | Non-SS | 59 | F | White | - | - | 0 |
| GRU-1622(S23) | Non-SS | 35 | F | African American | - | - | 0 |
| p1034411-7(S24) | Non-SS | 55 | F | White/Native American | - | - | 0 |
|  |  |  |  |  |  |  |  |
| GRU-0349(S25) | SS | 63 | F | White | - | - | 2 |
| GRU-0944(S26) | SS | 54 | F | White | + | + | 3 |
| GRU-0943(S27) | SS | 46 | F | White | - | - | 3 |
| p1001373-1(S28) | SS | 58 | F | Native American | + | + | 10.6 |
