# Supplementary material for: Transcriptomic and Network Analysis of Minor Salivary Glands of Patients With Primary Sjögren’s Syndrome
Source: Front Immunol. 2021 Jan 8;11:606268. doi: 10.3389/fimmu.2020.606268 (PMC7821166; doi:10.3389/fimmu.2020.606268)
Supplement: Supplementary file 6 [file Table_6.docx]

| Supplementary Table S6 | |
| --- | --- |
| **Term** | **Associated Enriched Genes** |
| Immune Response | GPR183, CR2, GZMA, CCR1, CXCL9, CCL19, HLA-DMB, CXCL11, IL7R, AIM2, CXCL10, HLA-F, CCR7, CXCL13, ICOS, IRF8, HLA-DPA1, LTB, HLA-DRA |
| Type I Interferon Signaling Pathway | IFIT3, IFI27, IFITM1, IRF8, XAF1, MX1, STAT1, HLA-F |
| Defense Response to Virus | IFIT3, NLRC5, PTPRC, IFITM1, CXCL9, IFI44L, MX1, STAT1, GBP1, CXCL10 |
| Chemokine-Mediated Signaling Pathway | CXCR4, CXCL13, CCR1, CXCL9, CCL19, CXCL11, CXCL10 |
| Dendritic Cell Chemotaxis | GPR183, CCR7, CXCR4, CCR1, CCL19 |
| Chemotaxis | DOCK2, CCR7, RAC2, CXCR4, CCR1, CXCL9, CXCL11, CXCL10 |
| Inflammatory Response | CYBB, CCR7, GBP5, CXCR4, CXCL13, CCR1, CXCL9, CCL19, CXCL11, AIM2, CXCL10 |
| Interferon-Gamma-Mediated Signaling Pathway | IRF8, HLA-DPA1, STAT1, GBP1, HLA-DRA, HLA-F |
| T Cell Costimulation | CD3D, ICOS, LCK, CCL19, HLA-DPA1, HLA-DRA |
| Cell Surface Receptor Signaling Pathway | PTPRC, CD3D, IFITM1, CXCL13, CCR1, CD2, IL7R, CXCL10 |
| Leukocyte Migration | CD48, SELL, MMP9, LCK, CD2, ITGA4 |
| Defense Response | CD48, TAP1, CXCL9, HCP5, MX1 |
| Establishment of T Cell Polarity | DOCK2, CCR7, CCL19 |
| Positive Regulation of Camp Metabolic Process | CXCL9, CXCL11, CXCL10 |
| T Cell Differentiation | PTPRC, CD3D, LCK, IL7R |
