# Supplementary material for: Transcriptomic and Network Analysis of Minor Salivary Glands of Patients With Primary Sjögren’s Syndrome
Source: Front Immunol. 2021 Jan 8;11:606268. doi: 10.3389/fimmu.2020.606268 (PMC7821166; doi:10.3389/fimmu.2020.606268)
Supplement: Supplementary file 7 [file Table_7.docx]

| Supplementary Table S7 | |
| --- | --- |
| **IPA Pathway** | **Associated Genes**  Green = Upregulated  Red = Downregulated |
| Th1 Pathway | PIK3C2G, CCR5, CD247, CD274, CD28, CD3D, CD3E, CD3G, CD4, CD40, CD40LG, CD80, CD86, CD8A, CXCR3, HAVCR2, HLA-A, HLA-B, HLA-DMA, HLA-DMB, HLA-DOA, HLA-DOB, HLA-DPA1, HLA-DPB1, HLA-DQA1, HLA-DQA2, HLA-DQB2, HLA-DRA, HLA-DRB1, HLA-DRB5, ICAM1, ICOS, ICOSLG, IFNG, IL10, IL10RA, IL12A, IL12B, IL12RB1, IL12RB2, IL18, IL18R1, IL2, IL27RA, IL6, IL6R, IRF1, ITGB2, JAK2, JAK3, LGALS9, LTA, MIR146A, NFATC2, NOTCH3, PIK3C2B, PIK3CD, PIK3CG, PIK3R5, PIK3R6, PRKCQ, RUNX3, SOCS1, SOCS3, STAT1, STAT4, TBX21, TNFSF11, TYK2, VAV1 |
| Th2 Pathway | ACVR1C, PIK3C2G, CCR1, CCR3, CCR4, CCR5, CCR8, CD247, CD28, CD3D, CD3E, CD3G, CD4, CD40, CD80, CD86, CXCR4, CXCR6, GFI1, HAVCR1, HLA-A, HLA-B, HLA-DMA, HLA-DMB, HLA-DOA, HLA-DOB, HLA-DPA1, HLA-DPB1, HLA-DQA1, HLA-DQA2, HLA-DQB2, HLA-DRA, HLA-DRB1, HLA-DRB5, ICAM1, ICOS, ICOSLG, IFNG, IKZF1, IL10, IL12A, IL12B, IL12RB1, IL12RB2, IL2, IL2RA, IL2RB, IL2RG, IL4R, ITGB2, JAK2, JAK3, NFATC2, NOTCH3, PIK3C2B, PIK3CD, PIK3CG, PIK3R5, PIK3R6, PRKCQ, RUNX3, S1PR1, SOCS3, SPI1, STAT4, STAT5A, TBX21, TGFB1, TIMD4, TNFRSF4, TNFSF4, TYK2, VAV1 |
| Dendritic Cell Maturation | CREB3L4, PDIA3, PIK3C2G, PLCB4, B2M, CCR7, CD1A, CD1B, CD1C, CD1D, CD40, CD40LG, CD80, CD83, CD86, COL18A1, FCER1G, FCGR1A, FCGR1B, FCGR2A, FCGR2B, FCGR2C, FSCN1, HLA-A, HLA-B, HLA-C, HLA-DMA, HLA-DMB, HLA-DOA, HLA-DOB, HLA-DQA1, HLA-DRA, HLA-DRB1, HLA-DRB5, HLA-E, HLA-F, ICAM1, IKBKB, IKBKE, IL10, IL12A, IL12B, IL15, IL18, IL1B, IL1RN, IL23A, IL32, IL6, IRF8, JAK2, LTA, LTB, LY75, MAP3K14, MAPK11, MAPK12, MYD88, NFKB2, NFKBIA, NFKBID, NFKBIE, PIK3C2B, PIK3CD, PIK3CG, PIK3R5, PIK3R6, PLCB2, PLCG1, PLCG2, PLCH1, PLCL2, RELB, STAT1, STAT2, STAT4, TLR9, TNF, TNFRSF1B, TYROBP, ZBTB12 |
| Systemic Lupus Erythematosus In B Cell Signaling Pathway | CALM1, EDA, PIK3C2G, PPP3CA, PRKCI, YES1, BCL2, BLNK, BTK, CAMK4, CARD11, CBL, CD19, CD22, CD40, CD40LG, CD5, CD70, CD72, CD79A, CD79B, CSK, FASLG, FCGR2A, FCGR2B, FCGR2C, FGR, FOS, FYN, HCK, IFIH1, IFIT2, IFIT3, IFNAR2, IFNG, IL10, IL12A, IL12B, IL15, IL18, IL1B, IL2, IL21, IL6, IL6R, IRF3, IRF5, IRF7, IRF9, ISG15, ISG20, JAK2, LCK, LILRB3, LILRB4, LTA, LTB, MAP3K14, MCL1, MYC, MYD88, NFATC2, NFKB2, PAG1, PDCD1, PIK3C2B, PIK3CD, PIK3CG, PIK3R5, PIK3R6, PIM2, PLCG2, PRKCB, PRKCD, PRKCG, PRKCQ, PTPN6, RAC2, RAP2B, RARRES3, RASGRP1, RASGRP2, STAT1, STAT2, SYK, TGFB1, TLR7, TLR8, TLR9, TMEM173, TNF, TNFRSF13C, TNFSF11, TNFSF13B, TNFSF14, TNFSF4, TNFSF8, TNFSF9, TRAF1, TRAF2, TRAF3, TRAF5, TYK2, VAV1 |
| PD-1, PD-L1 cancer immunotherapy pathway | PIK3C2G, B2M, CD247, CD274, CD28, CD80, CSK, FOXP3, HLA-A, HLA-B, HLA-C, HLA-DMA, HLA-DMB, HLA-DOA, HLA-DOB, HLA-DPA1, HLA-DPB1, HLA-DQA1, HLA-DQA2, HLA-DQB2, HLA-DRA, HLA-DRB1, HLA-DRB5, HLA-E, HLA-F, HLA-G, IFNG, IL12A, IL12B, IL2, IL2RA, IL2RB, IL2RG, JAK2, JAK3, KIAA1524, LAT, LCK, LCP2, MR1, PDCD1, PDCD1LG2, PIK3C2B, PIK3CD, PIK3CG, PIK3R5, PIK3R6, PLCG1, PRKCQ, RASGRP1, STAT5A, TGFB1, TNF, TNFRSF1B, TYK2, ZAP70, ZBTB12 |
| Neuroinflammation Signaling Pathway | ACVR1C, APP, BACE2, CREB3L4, GABRB1, GABRG3, MAPK4, MAPK6, PIK3C2G, PLA2G12A, PLA2G4A, PPP3CA, SNCA, AGER, B2M, BCL2, BIRC3, BIRC5, CASP1, CASP8, CCL2, CCL3, CCL5, CD200, CD200R1, CD40, CD80, CD86, CSF1R, CX3CL1, CXCL10, CYBB, FAS, FASLG, FOS, GABRR2, GLS, HLA-A, HLA-B, HLA-C, HLA-DMA, HLA-DMB, HLA-DOA, HLA-DOB, HLA-DQA1, HLA-DRA, HLA-DRB1, HLA-DRB5, HLA-E, HLA-F, HMOX1, ICAM1, IFNG, IKBKB, IKBKE, IL10, IL12A, IL12B, IL18, IL1B, IL6, IL6R, IRAK2, IRF3, IRF7, JAK2, JAK3, KCNJ5, MAPK11, MAPK12, MMP9, MYD88, NAIP, NCF1, NCF2, NFATC2, NFKB2, NLRP3, P2RX7, PIK3C2B, PIK3CD, PIK3CG, PIK3R5, PIK3R6, PLA2G2D, PLA2G4C, PLCG1, PLCG2, PRKCG, PYCARD, SLC1A3, SLC6A12, STAT1, SYK, TGFB1, TLR1, TLR10, TLR6, TLR7, TLR8, TLR9, TNF, TRAF3, TYK2, TYROBP, VCAM1, ZBTB12 |
| T Cell Exhaustion Signaling Pathway | ACVR1C, PIK3C2G, PPM1L, PPP2R3A, BATF, BTLA, CD274, CD28, CD80, CD86, CTLA4, EOMES, FCER1G, FOS, GZMB, HAVCR2, HLA-A, HLA-B, HLA-C, HLA-DMA, HLA-DMB, HLA-DOA, HLA-DOB, HLA-DPA1, HLA-DPB1, HLA-DQA1, HLA-DQA2, HLA-DQB2, HLA-DRA, HLA-DRB1, HLA-DRB5, HLA-E, HLA-F, HLA-G, IFNAR2, IFNG, IL10, IL10RA, IL12A, IL12B, IL12RB1, IL12RB2, IL6, IL6R, IRF4, IRF9, JAK2, JAK3, LAG3, LGALS9, MAPK12, NFATC2, PDCD1, PDCD1LG2, PDK1, PIK3C2B, PIK3CD, PIK3CG, PIK3R5, PIK3R6, PLCG1, PLCG2, PPP2R2B, PRDM1, PRKCQ, PTPN6, RAP2B, STAT1, STAT2, STAT4, TBX21, TCF7, TGFB1, TNFRSF14, TYK2, ZAP70 |
| Crosstalk between Dendritic Cells and Natural Killer Cells | ACTG2, ACTB, CCR7, CD226, CD28, CD40, CD40LG, CD69, CD80, CD83, CD86, CSF2RB, FAS, FASLG, FSCN1, HLA-A, HLA-B, HLA-C, HLA-DRA, HLA-DRB1, HLA-DRB5, HLA-E, HLA-F, HLA-G, ICAM3, IFNG, IL12A, IL12B, IL15, IL15RA, IL18, IL2, IL2RB, IL2RG, IL3RA, IL6, ITGAL, KLRK1, LTA, LTB, MICB, NCR3, NFKB2, PRF1, TLN1, TLR7, TLR9, TNF, TNFRSF1B, TYROBP |
| iCOS-iCOSL Signaling in T Helper Cells | CALM1, PIK3C2G, PPP3CA, CAMK4, CD247, CD28, CD3D, CD3E, CD3G, CD4, CD40, CD40LG, CD80, CD86, CSK, FCER1G, GRAP2, HLA-A, HLA-B, HLA-DMA, HLA-DMB, HLA-DOA, HLA-DOB, HLA-DQA1, HLA-DRA, HLA-DRB1, HLA-DRB5, ICOS, ICOSLG, IKBKB, IKBKE, IL2, IL2RA, IL2RB, IL2RG, ITK, LAT, LCK, LCP2, NFATC2, NFKB2, NFKBIA, NFKBID, NFKBIE, PIK3C2B, PIK3CD, PIK3CG, PIK3R5, PIK3R6, PLCG1, PLEKHA2, PRKCQ, PTPRC, TRAT1, VAV1, ZAP70 |
| Type I Diabetes Mellitus Signaling | CPE, ICA1, SOCS6, BCL2, CASP8, CD247, CD28, CD3D, CD3E, CD3G, CD80, CD86, FAS, FASLG, FCER1G, GZMB, HLA-A, HLA-B, HLA-C, HLA-DMA, HLA-DMB, HLA-DOA, HLA-DOB, HLA-DQA1, HLA-DRA, HLA-DRB1, HLA-DRB5, HLA-E, HLA-F, HLA-G, IFNG, IKBKB, IKBKE, IL12A, IL12B, IL1B, IL2, IRF1, JAK2, LTA, MAP3K14, MAP3K5, MAPK11, MAPK12, MYD88, NFKB2, NFKBIA, NFKBID, NFKBIE, PRF1, SOCS1, SOCS3, STAT1, TNF, TNFRSF1B, TRAF2 |
| Natural Killer Cell Signaling | HSPA2, MAP3K15, PIK3C2G, PVR, WASL, B2M, CD226, CD244, CD247, CD48, COL18A1, FASLG, FCER1G, FCGR2A, FYN, HCST, HLA-A, HLA-B, HLA-C, HLA-E, HLA-F, HLA-G, IFNG, IL12A, IL12B, IL12RB1, IL12RB2, IL15, IL18, IL18R1, IL18RAP, IL2, IL2RB, ITGAL, JAK2, JAK3, KLRB1, KLRK1, LAIR1, LAT, LCK, LCP2, LILRB1, MAP3K14, MAP3K5, MAP3K8, MAPK11, MAPK12, MICB, MYD88, NCR1, NCR3, NFATC2, NFKB2, PIK3C2B, PIK3CD, PIK3CG, PIK3R5, PIK3R6, PLCG1, PLCG2, PRKCQ, PTK2B, PTPN6, RAC2, RAP2B, SH2D1A, SIGLEC7, STAT4, SYK, TYK2, TYROBP, VAV1, WAS, WIPF1, ZAP70 |
| CD28 Signaling in T Helper Cells | CALM1, PIK3C2G, PPP3CA, ARPC1B, CAMK4, CARD11, CD247, CD28, CD3D, CD3E, CD3G, CD4, CD80, CD86, CSK, CTLA4, FCER1G, FOS, FYN, GRAP2, HLA-A, HLA-B, HLA-DMA, HLA-DMB, HLA-DOA, HLA-DOB, HLA-DQA1, HLA-DRA, HLA-DRB1, HLA-DRB5, IKBKB, IKBKE, IL2, ITK, LAT, LCK, LCP2, MAPK12, NFATC2, NFKB2, NFKBIA, NFKBID, NFKBIE, PIK3C2B, PIK3CD, PIK3CG, PIK3R5, PIK3R6, PLCG1, PRKCQ, PTPN6, PTPRC, SYK, VAV1, WAS, ZAP70 |
| Role of Pattern Recognition Receptors in Recognition of Bacteria and Viruses | EDA, PIK3C2G, PRKCI, C1QA, C1QB, C1QC, C3, C3AR1, C5AR1, CASP1, CCL5, CD40LG, CD70, CLEC6A, CLEC7A, DDX58, FASLG, IFIH1, IFNG, IL10, IL12A, IL12B, IL15, IL18, IL1B, IL2, IL21, IL6, IRF3, IRF7, LTA, LTB, MAPK12, MYD88, NFKB2, NLRC4, NLRP3, OAS1, OAS2, OAS3, PIK3C2B, PIK3CD, PIK3CG, PIK3R5, PIK3R6, PLCG2, PRKCB, PRKCD, PRKCG, PRKCQ, SYK, TGFB1, TLR1, TLR6, TLR7, TLR8, TLR9, TNF, TNFSF11, TNFSF13B, TNFSF14, TNFSF4, TNFSF8, TNFSF9 |
| Tec Kinase Signaling | ACTG2, FRK, GNAI1, GNAQ, GNAS, ITGA2, PIK3C2G, PRKCI, PTK2, RHOB, RHOU, RND2, RND3, YES1, ACTB, BLK, BTK, FAS, FASLG, FCER1G, FGR, FNBP1, FOS, FYN, GNAI2, GNG2, HCK, ITGA4, ITK, JAK2, JAK3, LCK, MAPK12, NFKB2, PIK3C2B, PIK3CD, PIK3CG, PIK3R5, PIK3R6, PLCG1, PLCG2, PRKCB, PRKCD, PRKCG, PRKCQ, PTK2B, RAC2, RHOBTB2, RHOF, RHOG, RHOH, STAT1, STAT2, STAT4, STAT5A, TNF, TNFRSF10A, TNFRSF10B, TNFRSF25, TXK, TYK2, VAV1, WAS |
| Role of NFAT in Regulation of the Immune Response | CALM1, GNAI1, GNAQ, GNAS, PIK3C2G, PLCB4, PPP3CA, AKAP5, BLNK, BTK, CAMK4, CD247, CD28, CD3D, CD3E, CD3G, CD4, CD79A, CD79B, CD80, CD86, FCER1G, FCGR1A, FCGR1B, FCGR2A, FCGR2B, FCGR2C, FOS, FYN, GNAI2, GNG2, HLA-A, HLA-B, HLA-DMA, HLA-DMB, HLA-DOA, HLA-DOB, HLA-DQA1, HLA-DRA, HLA-DRB1, HLA-DRB5, IKBKB, IKBKE, ITK, LAT, LCK, LCP2, MEF2C, NFATC2, NFKB2, NFKBIA, NFKBID, NFKBIE, PIK3C2B, PIK3CD, PIK3CG, PIK3R5, PIK3R6, PLCB2, PLCG1, PLCG2, PRKCQ, RAP2B, SYK, ZAP70 |
| PKC-Theta Signaling in T Lymphocytes | CACNA1D, CACNB4, MAP3K15, PIK3C2G, PPP3CA, CACNA1F, CARD11, CD247, CD28, CD3D, CD3E, CD3G, CD4, CD80, CD86, FCER1G, FOS, FYN, GRAP2, HLA-A, HLA-B, HLA-DMA, HLA-DMB, HLA-DOA, HLA-DOB, HLA-DQA1, HLA-DRA, HLA-DRB1, HLA-DRB5, IKBKB, IKBKE, IL2, LAT, LCK, LCP2, MAP3K14, MAP3K5, MAP3K8, NFATC2, NFKB2, NFKBIA, NFKBID, NFKBIE, PIK3C2B, PIK3CD, PIK3CG, PIK3R5, PIK3R6, PLCG1, PLCG2, PRKCQ, RAC2, RAP2B, VAV1, ZAP70 |
| TREM1 Signaling | CASP1, CCL2, CCL3, CD40, CD83, CD86, CIITA, FCGR2B, ICAM1, IL10, IL18, IL1B, IL6, ITGAX, JAK2, LAT2, MYD88, NFKB2, NLRC3, NLRC4, NLRC5, NLRP14, NLRP3, PLCG1, PLCG2, SIGIRR, STAT5A, TLR1, TLR10, TLR6, TLR7, TLR8, TLR9, TNF, TYROBP |
| Cardiac Hypertrophy Signaling | ACVR1C, ADCY2, CACNA1D, CALM1, EDA, EIF4EBP1, FGF10, FGF12, FGF13, FGF14, FGFRL1, GNAI1, GNAQ, GNAS, IL13RA2, IL17RD, IL20RA, ITGA2, MAP3K15, MPPED2, PDE3B, PDE4D, PDE8B, PDIA3, PIK3C2G, PLCB4, PLD6, PPP3CA, PRKACB, PRKAR2B, PRKCI, PTK2, ADCY1, ADCY7, ADRA2A, ADRB2, CAMK4, CD40LG, CD70, CSF2RB, CYBB, DIAPH3, FASLG, FGF11, GNAI2, GNG2, HDAC10, HDAC9, IFNG, IKBKB, IKBKE, IL10RA, IL12A, IL12B, IL12RB1, IL12RB2, IL15, IL15RA, IL17RA, IL18, IL18R1, IL18RAP, IL1B, IL1R2, IL2, IL21, IL21R, IL22RA2, IL27RA, IL2RA, IL2RB, IL2RG, IL3RA, IL4R, IL6, IL6R, IL7R, IL9R, ITGA4, JAK2, LTA, LTB, MAP3K14, MAP3K5, MAP3K8, MAPK11, MAPK12, MEF2C, MYC, NFATC2, NFKB2, PDE6B, PDE6G, PDE7A, PDK1, PIK3C2B, PIK3CD, PIK3CG, PIK3R5, PIK3R6, PLCB2, PLCG1, PLCG2, PLCH1, PLCL2, PRKCB, PRKCD, PRKCG, PRKCQ, RAP2B, TGFB1, TNF, TNFRSF1B, TNFSF11, TNFSF13B, TNFSF14, TNFSF4, TNFSF8, TNFSF9, WNT10A, WNT10B, WNT16 |
| Leukocyte Extravasation Signaling | ACTG2, ARHGAP12, ARHGAP5, CLDN12, GNAI1, ITGA2, PIK3C2G, PRKCI, PTK2, TIMP1, WASL, ACTB, ARHGAP4, ARHGAP9, BTK, CDH5, CLDN15, CXCR4, CYBA, CYBB, GNAI2, ICAM1, ICAM3, ITGA4, ITGAL, ITGAM, ITGB2, ITK, MAPK11, MAPK12, MMP1, MMP11, MMP12, MMP25, MMP9, MSN, NCF1, NCF2, NCF4, PECAM1, PIK3C2B, PIK3CD, PIK3CG, PIK3R5, PIK3R6, PLCG1, PLCG2, PRKCB, PRKCD, PRKCG, PRKCQ, PTK2B, RAC2, RASGRP1, RHOH, SELPLG, SIPA1, SPN, THY1, TXK, VAV1, VCAM1, WAS, WIPF1 |
| Production of Nitric Oxide and Reactive Oxygen Species in Macrophages | APOD, MAP3K15, PCYOX1, PIK3C2G, PPM1L, PPP2R3A, PRKCI, RHOB, RHOU, RND2, RND3, APOC1, APOE, APOL1, CYBA, CYBB, FNBP1, FOS, IFNG, IKBKB, IKBKE, IRF1, IRF8, JAK2, JAK3, MAP3K14, MAP3K5, MAP3K8, MAPK11, MAPK12, NCF1, NCF2, NCF4, NFKB2, NFKBIA, NFKBID, NFKBIE, PIK3C2B, PIK3CD, PIK3CG, PIK3R5, PIK3R6, PLCG1, PLCG2, PPP2R2B, PRKCB, PRKCD, PRKCG, PRKCQ, PTPN6, RAC2, RHOBTB2, RHOF, RHOG, RHOH, SAA4, SPI1, STAT1, TNF, TNFRSF1B, TYK2 |
| OX40 Signaling Pathway | B2M, BCL2, CD247, CD3D, CD3E, CD3G, CD4, FCER1G, HLA-A, HLA-B, HLA-C, HLA-DMA, HLA-DMB, HLA-DOA, HLA-DOB, HLA-DPA1, HLA-DPB1, HLA-DQA1, HLA-DQA2, HLA-DQB2, HLA-DRA, HLA-DRB1, HLA-DRB5, HLA-E, HLA-F, HLA-G, IL2, MAPK12, NFKB2, NFKBIA, NFKBID, NFKBIE, TNFRSF4, TNFSF4, TRAF2, TRAF3, TRAF5 |
