# Supplementary material for: Transcriptomic and Network Analysis of Minor Salivary Glands of Patients With Primary Sjögren’s Syndrome
Source: Front Immunol. 2021 Jan 8;11:606268. doi: 10.3389/fimmu.2020.606268 (PMC7821166; doi:10.3389/fimmu.2020.606268)
Supplement: Supplementary file 8 [file DataSheet_1.docx]

**Supplementary Figure S1.** **Integrated Analysis**. Venn diagram showing the overlay of down-regulated genes identified in the RNA-seq dataset described here (Oyelakin et al.) and Min et al. (1) and Liu et al. (2) datasets.

**Supplementary Figure S2.** **Validation of Select Genes**. Quantitative RT-PCR analysis validating the mRNA expression levels of a panel of select genes in non-pSS controls and pSS patient samples that were subjected to RNA-seq analysis (see Materials and Methods section for patient sample IDs). Values were normalized to the housekeeping gene GAPDH. Data are represented as ± S.D (n=3). *p<0.1, **p<0.01, ***p<0.001

**Supplementary Figure S3.** **Validation of Select Genes**. Quantitative RT-PCR analysis validating the mRNA expression levels of a panel of select genes in a cohort of non-pSS controls and pSS patient samples that were not subjected to RNA-seq analysis (see Materials and Methods Section for patient sample IDs). Values were normalized to the housekeeping gene GAPDH. Data are represented as ± S.D (n=4). **p<0.01

**References**

1. Min HK, Moon SJ, Park KS, Kim KJ. Integrated systems analysis of salivary gland transcriptomics reveals key molecular networks in Sjogren's syndrome. Arthritis Res Ther. 2019;21(1):294.

2. Liu Z, Li F, Pan A, Xue H, Jiang S, Zhu C, et al. Elevated CCL19/CCR7 Expression During the Disease Process of Primary Sjogren's Syndrome. Front Immunol. 2019;10:795.
